# A p53/lnc‐Ip53 Negative Feedback Loop Regulates Tumor Growth and Chemoresistance

**DOI:** 10.1002/advs.202001364

**Published:** 2020-09-28

**Authors:** Li‐Zhen Zhang, Jin‐E Yang, Yu‐Wei Luo, Feng‐Ting Liu, Yun‐Fei Yuan, Shi‐Mei Zhuang

**Affiliations:** ^1^ MOE Key Laboratory of Gene Function and Regulation School of Life Sciences Collaborative Innovation Center for Cancer Medicine Sun Yat‐sen University Guangzhou 510275 China; ^2^ Department of Hepatobilliary Oncology Cancer Center Sun Yat‐sen University Guangzhou 510060 China; ^3^ Key Laboratory of Liver Disease of Guangdong Province The Third Affiliated Hospital Sun Yat‐sen University Guangzhou 510630 China

**Keywords:** acetylation, lnc‐Ip53, non‐coding RNA, p53, tumor growth

## Abstract

Acetylation is a critical mechanism to modulate tumor‐suppressive activity of p53, but the causative roles of long non‐coding RNAs (lncRNAs) in p53 acetylation and their biological significance remain unexplored. Here, lncRNA LOC100294145 is discovered to be transactivated by p53 and is thus designated as lnc‐Ip53 for lncRNA induced by p53. Furthermore, lnc‐Ip53 impedes p53 acetylation by interacting with histone deacetylase 1 (HDAC1) and E1A binding protein p300 (p300) to prevent HDAC1 degradation and attenuate p300 activity, resulting in abrogation of p53 activity and subsequent cell proliferation and apoptosis resistance. Mouse xenograft models reveal that lnc‐Ip53 promotes tumor growth and chemoresistance in vivo, which is attenuated by an HDAC inhibitor. Silencing lnc‐Ip53 inhibits the growth of xenografts with wild‐type p53, but not those expressing acetylation‐resistant p53. Consistently, lnc‐Ip53 is upregulated in multiple cancer types, including hepatocellular carcinoma (HCC). High levels of lnc‐Ip53 is associated with low levels of acetylated p53 in human HCC and mouse xenografts, and is also correlated with poor survival of HCC patients. These findings identify a novel p53/lnc‐Ip53 negative feedback loop in cells and indicate that abnormal upregulation of lnc‐Ip53 represents an important mechanism to inhibit p53 acetylation/activity and thereby promote tumor growth and chemoresistance, which may be exploited for anticancer therapy.

## Graphical abstract

Acetylation is indispensable for activation of tumor suppressor p53. LncRNA lnc‐Ip53 is induced by p53 and in turn represses p53 acetylation by interacting with HDAC1 and p300 to prevent HDAC1 degradation and attenuate p300 activity, which consequently abrogates p53 activity and thus facilitates cell proliferation and apoptosis resistance. Abnormal upregulation of lnc‐Ip53 in tumors promotes tumor growth and confers chemoresistance.

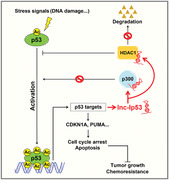

## 1 Introduction

The tumor suppressor p53 is a central regulator in cell cycle and apoptosis, and functional loss of p53 remains the most common event in cancers, which contributes to tumor growth and chemoresistance.^[^ ^1^, ^2^ ^]^ In response to various cellular stresses, including DNA damage, oxidative stress, or oncogene activation, p53 is upregulated/activated, then acts as a transcription factor to transactivate downstream target genes, like cyclin‐dependent kinase inhibitor 1A (CDKN1A), p53‐upregulated modulator of apoptosis (PUMA) and BCL2 associated X (BAX), thereby triggers cell cycle arrest and/or apoptosis. Because of its importance in physiological and pathological processes, p53 activity is tightly regulated by multiple mechanisms, especially post‐translational modifications.^[^ ^2^ ^]^

Acetylation is an indispensable modification mechanism to activate p53 during stress responses. The acetylation state of a protein is controlled by histone acetyltransferases (HAT) and histone deacetylases (HDAC), enzymes that catalyze the addition and removal of an acetyl group from a lysine residue, respectively. Specially, p53 is acetylated by E1A binding protein p300 (p300)/CREB binding protein (CBP) at lysines 164, 370, 372, 373, 381, 382, and 386, by MOF/Tat interactive protein 60 kDa (TIP60) at lysine 120, and by monocytic leukemia zinc finger (MOZ) at lysines 120 and 382, and it is deacetylated by HDAC1 and sirtuin 1 (SIRT1).^[^ ^2^ ^]^ Acetylated p53 displays decreased ubiquitylation, enhanced DNA binding activity, and thus increased transcriptional activity.^[^ ^3^, ^4^ ^]^ Loss of acetylation at all eight lysines completely abolishes p53‐mediated cell cycle arrest and apoptosis.^[^ ^5^ ^]^ The p53^KQ/KQ^ knock‐in mice that express acetylation‐mimicking form of p53 show neonatal lethality with substantial p53 activation and induction of different p53 target genes,^[^ ^6^ ^]^ further confirming the importance of acetylation for p53 activation in vivo.

Long non‐coding RNAs (lncRNAs) belong to a class of non‐coding transcripts with more than 200 nucleotides and they may regulate various physiological and pathological processes by interacting with DNA, RNA, or proteins.^[^ ^7^ ^]^ Recently, lncRNAs emerge as p53‐responsive genes that mediate p53 function.^[^ ^8^, ^9^, ^10^, ^11^, ^12^, ^13^ ^]^ Moreover, a few lncRNAs have been shown to form positive or negative feedback loops to amplify or terminate p53 signaling.^[^ ^9^, ^11^, ^12^ ^]^ For example, in response to DNA damage, lncRNA DINO is induced by p53 and then binds to p53 to enhance its stabilization via unknown mechanism.^[^ ^11^ ^]^ Conversely, p53‐transactivated lincRNA‐RoR suppresses stress‐induced p53 translation by associating with heterogeneous nuclear ribonucleoprotein I (hnRNP I), and another p53‐reponsive lncRNA PURPL reduces basal levels of p53 protein by preventing MYB binding protein 1a (MYBBP1A)‐mediated p53 stabilization.^[^ ^9^, ^12^ ^]^ Nevertheless, the causative roles of lncRNAs, especially p53‐regulated lncRNAs, in p53 acetylation and their biological significance remain unexplored.

Here, we identify a lncRNA that is induced by p53 (named lnc‐Ip53) and then inhibits p53 acetylation by upregulating HDAC1 expression and attenuating p300 activity, which forms a negative feedback loop. Both in vitro and in vivo studies show that aberrant expression of lnc‐Ip53, which is frequently observed in hepatocellular carcinoma (HCC) and other cancer types, promotes tumor growth and chemoresistance by inhibiting p53 acetylation/activity.

## 2 Results

### 2.1 Lnc‐Ip53 Is a Direct Transcriptional Target of p53

**Figure 1:**
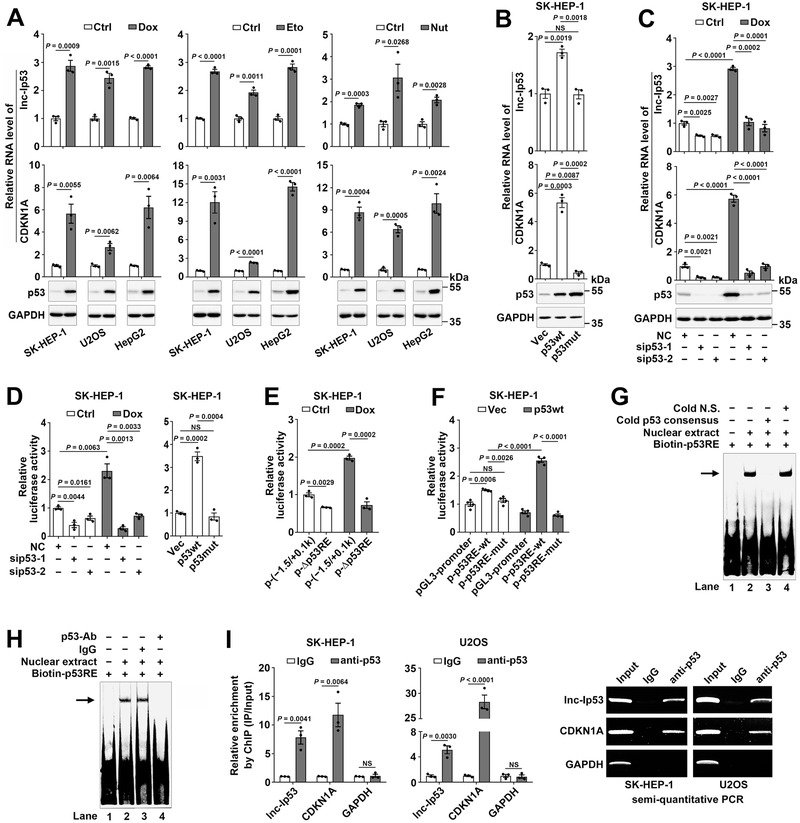
Lnc‐Ip53 is a transcriptional target of p53. A) Various p53 activators induced the expression of lnc‐Ip53 along with CDKN1A and p53. Cells were treated for 12 h with 0.5 µM doxorubicin (Dox), 50 µM etoposide (Eto), 10 µM nutlin‐3a (Nut), or vehicle control (Ctrl: PBS as control for Dox, DMSO as control for Eto and Nut). B) Overexpression of wild‐type p53 (p53wt) but not R175H mutant p53 (p53mut) increased the lnc‐Ip53 level. Cells stably expressing p53wt, p53mut, or control vector (Vec) were used. C) Silencing p53 (sip53) decreased the lnc‐Ip53 level. Cells were transfected with the indicated RNA duplexes for 32 h, then incubated with PBS (Ctrl) or 0.5 µM Dox for 12 h. For (A–C), the mRNA level of CDKN1A was used as a positive control. D) sip53 reduced the activity of the lnc‐Ip53 promoter (left), whereas overexpressing p53wt but not p53mut increased its activity (right). E) Deletion of p53RE reduced the activity of the lnc‐Ip53 promoter. F) p53 enhanced the activity of pGL3‐promoter reporter containing wild‐type but not mutant p53RE. G,H) EMSA and antibody‐supershift assay verified the in vitro interaction of p53 with p53RE in the lnc‐Ip53 promoter. The biotin‐labeled DNA‐protein complexes are indicated by arrow. Cold N.S., nonspecific scrambled oligonucleotide. Nuclear extracts were from SK‐HEP‐1 cells. I) p53 interacted with the lnc‐Ip53 promoter in vivo. Cells were incubated with 0.5 µM Dox for 6 h before ChIP. The antibody‐precipitated DNAs were amplified by real‐time quantitative PCR (qPCR, left) and semi‐quantitative PCR for 35 cycles (right). The promoters of CDKN1A and GAPDH were used as positive and negative controls, respectively. + or −, cells with (+) or without (−) the indicated treatment. Data are shown as mean ± SEM of at least three independent experiments; *p*‐values were determined by unpaired Student′s *t*‐test; NS, not significant.

To identify p53‐regulated lncRNAs, we analyzed the Global Run‐On sequencing data (GSE53966) from HCT116‐p53^+/+^ cells treated with nutlin‐3a, a p53 activator. A novel lncRNA LOC100294145, which was upregulated in nutlin‐treated HCT116‐p53^+/+^ cells, located in intergenic region and harbored a p53 binding site, was selected for further investigation (Figure S1A and Table S1, Supporting Information). As shown, various p53 activators, including doxorubicin (Dox), etoposide, and nutlin‐3a, significantly increased the levels of LOC100294145 and the well‐known p53‐transactivated genes (CDKN1A, MIR34AHG) in all three examined cell lines that had wild‐type p53 (**Figure** **1**A; Figure S1B, Supporting Information). LOC100294145 was therefore named lnc‐Ip53 (lncRNA induced by p53). In support, overexpressing wild‐type but not mutant p53 upregulated lnc‐Ip53 (Figure 1B; Figure S1C, Supporting Information), whereas silencing p53 (sip53) decreased both basal and Dox‐induced lnc‐Ip53 expression (Figure 1C; Figure S1D, Supporting Information). Furthermore, Dox induced lnc‐Ip53 expression in HCT116‐p53^+/+^ cells, but not in p53‐null cell lines, like HCT116‐p53^−/−^ and Hep3B (Figure S1E,F, Supporting Information). These data indicate lnc‐Ip53 as a p53‐responsive lncRNA.

We characterized lnc‐Ip53 as a 3094‐nt polyadenylated RNA without protein‐coding capacity (Figure S1G–I, Supporting Information). Both chromatin immunoprecipitation (ChIP)‐sequencing data from ENCODE (Figure S1H, Supporting Information) and our luciferase reporter assay (Figure S1J, Supporting Information) revealed that the 1500‐bp region upstream of lnc‐Ip53 exhibited promoter activity, which was reduced by sip53 and enhanced by overexpressing wild‐type but not mutant p53 (Figure 1D; Figure S1K, Supporting Information). A p53‐responsive element (p53RE) was predicted within this region and its deletion (p‐∆p53RE) diminished the lnc‐Ip53 promoter activity (Figure 1E; Figure S1L, Supporting Information). Consistently, insertion of the wild‐type but not mutant p53RE upstream of the promoter of “firefly” luciferase gene increased luciferase activity, which was further enhanced by p53 overexpression (Figure 1F; Figure S1M, Supporting Information). Electrophoretic mobility shift assay (EMSA) and antibody‐supershift assay revealed an in vitro interaction between p53 and p53RE (Figure 1G,H). ChIP further confirmed their in vivo interaction (Figure 1I). These results suggest that p53 transactivates lnc‐Ip53 transcription by directly binding to the p53RE in the lnc‐Ip53 promoter.

### 2.2 Lnc‐Ip53 Represses the Acetylation and Transcriptional Activity of p53

**Figure 2:**
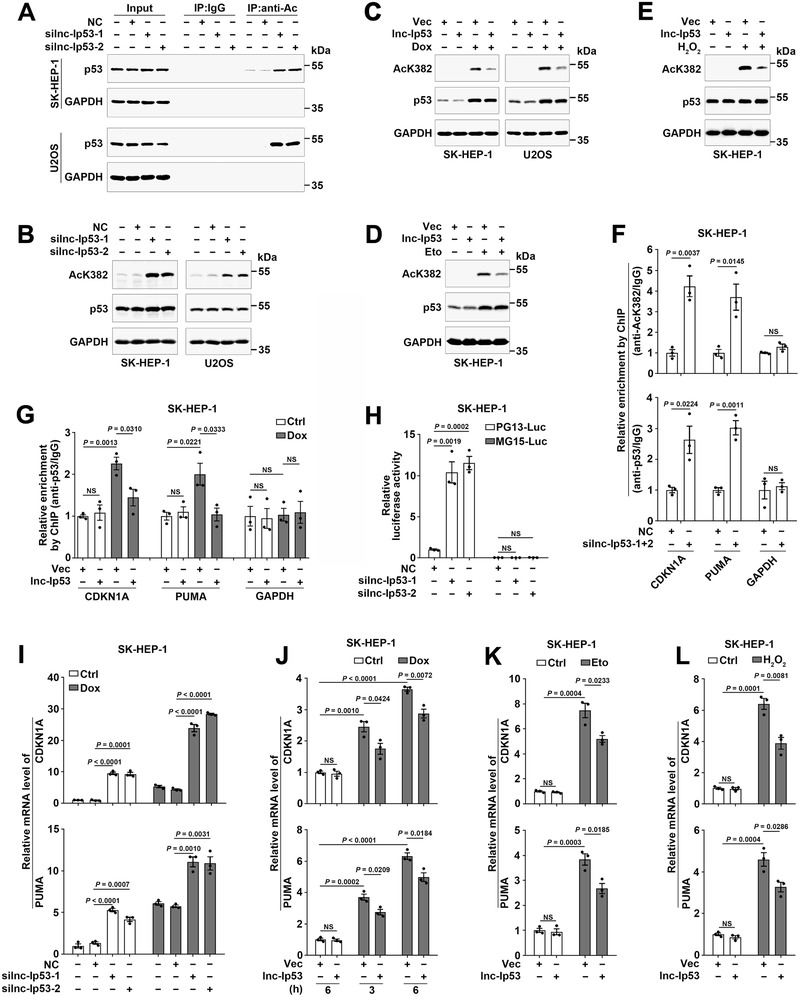
Lnc‐Ip53 diminishes the transcriptional activity of p53 by inhibiting its acetylation. A,B) Silencing lnc‐Ip53 (silnc‐Ip53) increased the levels of A) total acetylated p53 and B) K382‐acetylated p53. Cells were transfected with the indicated RNA duplexes for 42 h, then incubated with 5 µM SAHA for 6 h, followed by A) immunoprecipitation (IP) using IgG or antibody against acetyl lysines (anti‐Ac) and then immunoblotting for p53, or B) immunoblotting for K382‐acetylated p53 (AcK382). NC, negative control. silnc‐Ip53‐1 and silnc‐Ip53‐2, siRNA targeting different regions of lnc‐Ip53. C–E) Lnc‐Ip53 attenuated the DNA damage‐ and oxidation stress‐induced p53 acetylation at K382. Cells were incubated with C) 0.25 µM Dox or D) 50 µM Eto for 5 h, or E) 250 µM H_2_O_2_ for 12 h. F) silnc‐Ip53 increased the amount of CDKN1A and PUMA promoters that were precipitated by antibodies against K382‐acetylated (upper) or total p53 (lower). Cells were transfected with the indicated RNA duplexes for 48 h before ChIP. G) Lnc‐Ip53 attenuated the Dox‐promoted interaction between p53 and CDKN1A/PUMA promoters. Cells were incubated with PBS (Ctrl) or 0.25 µM Dox for 6 h before ChIP. For (F,G), the GAPDH promoter was used as a negative control. H) silnc‐Ip53 enhanced the activity of p53‐reponsive reporter. I) silnc‐Ip53 increased the mRNA levels of CDKN1A and PUMA. Cells were transfected with the indicated RNA duplexes for 46 h, then incubated with PBS or 0.25 µM Dox for 6 h, followed by qPCR. J–L) Lnc‐Ip53 attenuated the DNA damage‐ and oxidation stress‐triggered increase of CDKN1A and PUMA. Cells were incubated with J) PBS or 0.25 µM Dox for the indicated hours, or with K) 50 µM Eto for 6 h or L) 250 µM H_2_O_2_ for 12 h. Cells stably expressing lnc‐Ip53 or control vector (Vec) were used (C–E,G,J–L). + or −, cells with (+) or without (−) the indicated treatment. Data are shown as mean ± SEM of three independent experiments; *p*‐values were determined by unpaired Student′s *t*‐test; NS, not significant.

We next explored whether lnc‐Ip53 regulated the expression, protein modification, and activity of p53. Silencing lnc‐Ip53 (silnc‐Ip53) remarkably increased the levels of acetylated p53 (**Figure** **2**A; Figure S2A, Supporting Information). Examination on the representative site lysine 382 (K382) further confirmed elevation of K382‐acetylated p53 in lnc‐Ip53‐silencing cells (Figure 2B; Figure S2A–C, Supporting Information). However, neither the levels of total p53 and phosphorylated p53 nor its subcellular localization were affected by silnc‐Ip53 (Figure S2D–F, Supporting Information). Consistently, lnc‐Ip53 overexpression attenuated the DNA‐damaging (Dox or etoposide) and oxidation stress (H_2_O_2_) agent‐induced p53 acetylation (Figure 2C–E; Figure S2G, Supporting Information).

Acetylation of p53, which enhances DNA binding activity, is essential for its activity to transactivate downstream target genes, such as CDKN1A and PUMA.^[^ ^3^, ^4^, ^5^ ^]^ ChIP revealed that the amount of CDKN1A or PUMA promoters that were precipitated by antibodies against acetylated or total p53 significantly increased in lnc‐Ip53‐silencing cells (Figure 2F) but reduced in lnc‐Ip53‐overexpressing cells (Figure 2G). Moreover, silnc‐Ip53 significantly increased the luciferase activity of PG13‐Luc reporter that carried wild‐type p53 binding sites, but had no effect on the activity of MG15‐Luc that contained mutant p53 binding sites (Figure 2H; Figure S2H, Supporting Information). Consistently, silnc‐Ip53 enhanced the mRNA levels of CDKN1A and PUMA in p53‐wild‐type cells, but not in p53‐null cells (Figure 2I; Figure S2I,J, Supporting Information), whereas lnc‐Ip53 overexpression attenuated the Dox‐, etoposide‐ or H_2_O_2_‐induced upregulation of CDKN1A and PUMA (Figure 2J–L; Figure S2K, Supporting Information). Altogether, lnc‐Ip53 may impede p53 acetylation and then inhibit its activity.

**Figure 3:**
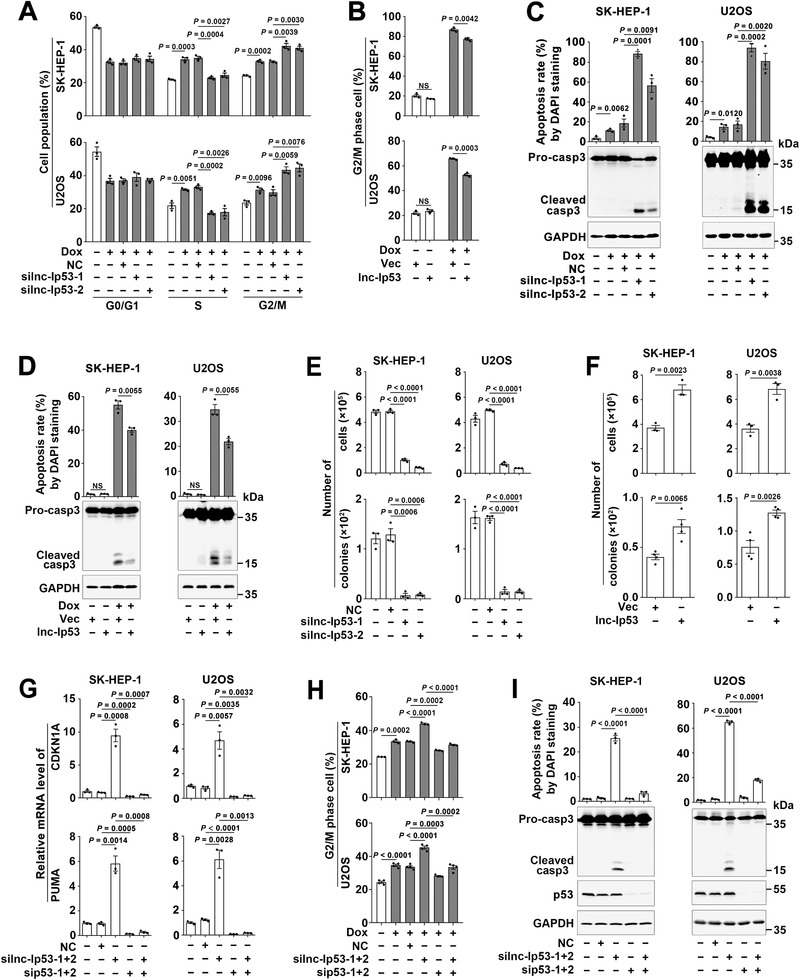
Lnc‐Ip53 impairs the p53‐mediated cell cycle arrest and apoptosis. A–D) Dox‐induced G2/M arrest and apoptosis were A,C) promoted by silnc‐Ip53 but B,D) attenuated by lnc‐Ip53 overexpression. For (A), cells were transfected with the indicated RNA duplexes for 39 h, then incubated with 0.1 µM Dox for 9 h before cell cycle analysis. For (B), cells were incubated with 0.1 µM Dox for 29 h (SK‐HEP‐1) or 26 h (U2OS) before cell cycle analysis. For (C), cells were transfected with the indicated RNA duplexes for 44 h, then incubated with 1 µM Dox for 28 h before 4′‐6′‐diamidino‐2‐phenylindole (DAPI) staining or 24 h before immunoblotting. For (D), cells were incubated with 1 µM Dox for 44 h before DAPI staining or 42 h before immunoblotting. E,F) Cell growth (upper) and colony formation (lower) in vitro were E) inhibited by silnc‐Ip53 but F) enhanced by lnc‐Ip53 overexpression. G–I) sip53 abrogated the roles of silnc‐Ip53 in G) enhancing CDKN1A/PUMA expression, and in H) promoting G2/M arrest and I) apoptosis. Cells were transfected with the indicated RNA duplexes G) for 52 h before qPCR, or H) for 39 h and then incubated with 0.1 µM Dox for 9 h before cell cycle analysis, or I) transfected with the indicated RNA duplexes for 72 h before DAPI staining or 68 h before immunoblotting. For (B,D,F), cells stably expressing lnc‐Ip53 or their controls (Vec) were used. + or −, cells with (+) or without (−) the indicated treatment. Data are shown as mean ± SEM of at least three independent experiments; *p*‐values were determined by unpaired Student′s *t*‐test; NS, not significant.

We then investigated whether lnc‐Ip53 influenced the p53‐regulated cell activities, such as cell cycle and apoptosis. As shown, Dox‐induced G2/M arrest was promoted by silnc‐Ip53 (**Figure** **3**A; Figure S3A, Supporting Information) but was diminished by lnc‐Ip53 overexpression (Figure 3B; Figure S3B, Supporting Information). In addition, silnc‐Ip53 promoted both basal and Dox‐induced apoptosis (Figure 3C; Figure S3C,D, Supporting Information), whereas lnc‐Ip53 overexpression attenuated Dox‐induced apoptosis (Figure 3D). Consistently, the growth and colony formation of tumor cells were significantly suppressed by silnc‐Ip53 (Figure 3E; Figure S3E, Supporting Information) but were promoted by lnc‐Ip53 overexpression (Figure 3F; Figure S3F, Supporting Information). Furthermore, sip53 antagonized the stimulatory effect of silnc‐Ip53 on the expression of CDKN1A and PUMA (Figure 3G) as well as G2/M arrest and apoptosis (Figure 3H,I). These results suggest that lnc‐Ip53 may attenuate cell cycle arrest and apoptosis by abating p53 activity.

### 2.3 Lnc‐Ip53 Promotes Tumor Growth and Chemoresistance by Impeding p53 Acetylation In Vivo

**Figure 4:**
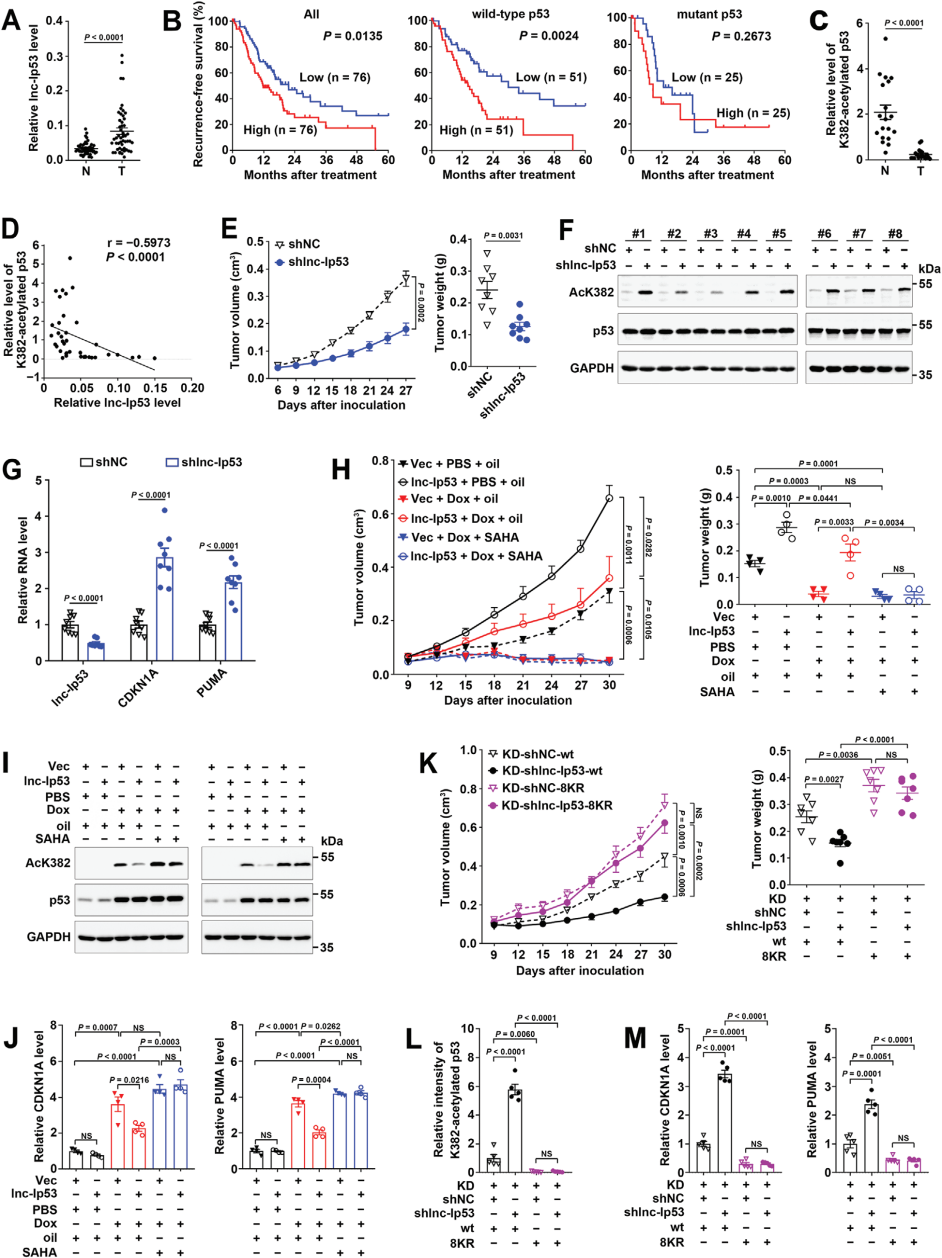
Lnc‐Ip53 promotes tumor growth and chemoresistance by impeding p53 acetylation in vivo. A) Lnc‐Ip53 was upregulated in human HCC. Lnc‐Ip53 expression was assessed by qPCR in 49 paired HCC (T) and adjacent non‐tumor liver (N) tissues. B) High lnc‐Ip53 level was associated with short recurrence‐free survival (RFS) of HCC with wild‐type but not mutant p53. Data were derived from TCGA. The median lnc‐Ip53 level was chosen as the cut‐off value for separating high‐lnc‐Ip53 group from low‐lnc‐Ip53 group. Survival curves were calculated using Kaplan–Meier method; *p*‐value was determined by log‐rank test. C,D) The level of K382‐acetylated p53 was C) reduced in HCC and D) inversely correlated with lnc‐Ip53 level. The data of 19 paired T and N tissues were derived from (A) and (C), and applied to Spearman′s correlation coefficient analysis (D). E–G) The xenografts with lnc‐Ip53 silencing displayed E) reduced growth, increase of F) acetylated‐p53 and G) CDKN1A/PUMA. SK‐HEP‐1 cells stably expressing shlnc‐Ip53 or control vector (shNC) were xenografted subcutaneously. *n*  =  8 mice per group. H–J) The xenografts with lnc‐Ip53 overexpression showed H) enhanced growth and chemoresistance, and decrease of I) acetylated‐p53 and J) CDKN1A/PUMA. SK‐HEP‐1 cells stably expressing lnc‐Ip53 or control vector (Vec) were xenografted subcutaneously. PBS and Oil, vehicle controls for Dox and SAHA, respectively. *n*  =  4 mice per group. K–M) Silencing lnc‐Ip53 K) repressed tumor growth and increased L) acetylated‐p53 and M) CDKN1A/PUMA in xenografts with wild‐type p53, but had no effect on those with acetylation‐resistant p53 (8KR). *n*  =  7 mice per group. Acetylated‐p53 was detected by immunoblotting (C,F,I) or immunohistochemistry (IHC; L); CDKN1A and PUMA were examined by qPCR (G,J,M). + or −, cells with (+) or without (−) the indicated treatment. Data are shown as mean ± SEM; *p*‐values were determined by paired (A,C) or unpaired (E,H,K, right; G,J–M) Student′s *t*‐test, or two‐way ANOVA (E,H,K, left); NS, not significant.

The in vivo biological significance of lnc‐Ip53 was then explored using human tumor tissues and mouse xenograft models. Analysis of our HCC study cohort and the transcriptome data from The Cancer Genome Atlas (TCGA) revealed that lnc‐Ip53 was upregulated in different malignancies, including HCC (**Figure** **4**A; Figure S4A, Supporting Information). The Kaplan–Meier survival analysis revealed an association between high lnc‐Ip53 level and short recurrence‐free survival (RFS) of HCC and this association was more pronounced among patients who carried wild‐type p53 (Figure 4B). Furthermore, the levels of acetylated p53 significantly decreased in HCC tissues (Figure 4C; Figure S4B, Supporting Information) and were inversely correlated with lnc‐Ip53 levels (Figure 4D), suggesting that upregulation of lnc‐Ip53 may contribute to reduction of acetylated p53 in cancers.

We further examined the in vivo effect of lnc‐Ip53 using mouse xenograft models. As shown, silencing lnc‐Ip53 significantly suppressed xenograft growth (Figure 4E; Figure S4C, Supporting Information) and increased the levels of acetylated p53 and CDKN1A/PUMA in xenografts (Figure 4F,G). On the other hand, enhanced lnc‐Ip53 expression allowed xenografts to grow faster and be more resistant to Dox treatment (Figure 4H; Figure S4D, Supporting Information), and to display lower levels of acetylated p53 and CDKN1A/PUMA (Figure 4I,J), whereas these effects of lnc‐Ip53 were attenuated by suberoylanilide hydroxamic acid (SAHA), a HDAC inhibitor that increases p53 acetylation and has been approved for anticancer therapy (Figure 4H–J; Figure S4D, Supporting Information).

To further validate whether lnc‐Ip53 exerted its function by regulating p53 acetylation, SK‐HEP‐1 cells with stable silencing of both p53 and lnc‐Ip53 (SK‐KD‐shlnc‐Ip53) or with stable silencing of p53 alone (SK‐KD‐shNC, negative control) were transfected with wild‐type p53 (SK‐KD‐shlnc‐Ip53‐wt, SK‐KD‐shNC‐wt) or acetylation‐resistant p53 that had lysine to arginine mutation in eight acetylation sites (SK‐KD‐shlnc‐Ip53‐8KR, SK‐KD‐shNC‐8KR). Compared with SK‐KD‐shNC‐wt‐derived xenografts, SK‐KD‐shlnc‐Ip53‐wt tumors displayed decrease of growth and increase of acetylated p53 and CDKN1A/PUMA levels (Figure 4K–M; Figure S4E,F, Supporting Information). Contrarily, no significant differences were observed between SK‐KD‐shNC‐8KR and SK‐KD‐shlnc‐Ip53‐8KR xenografts (Figure 4K–M; Figure S4E,F, Supporting Information), implying that silencing lnc‐Ip53 does not function in tumors with acetylation‐resistant p53.

Collectively, upregulation of lnc‐Ip53 may confer tumor with growth advantage and chemoresistance by inhibiting p53 acetylation.

### 2.4 Lnc‐Ip53 Attenuates p53 Acetylation by Interacting with HDAC1 to Increase Its Protein Stability

**Figure 5:**
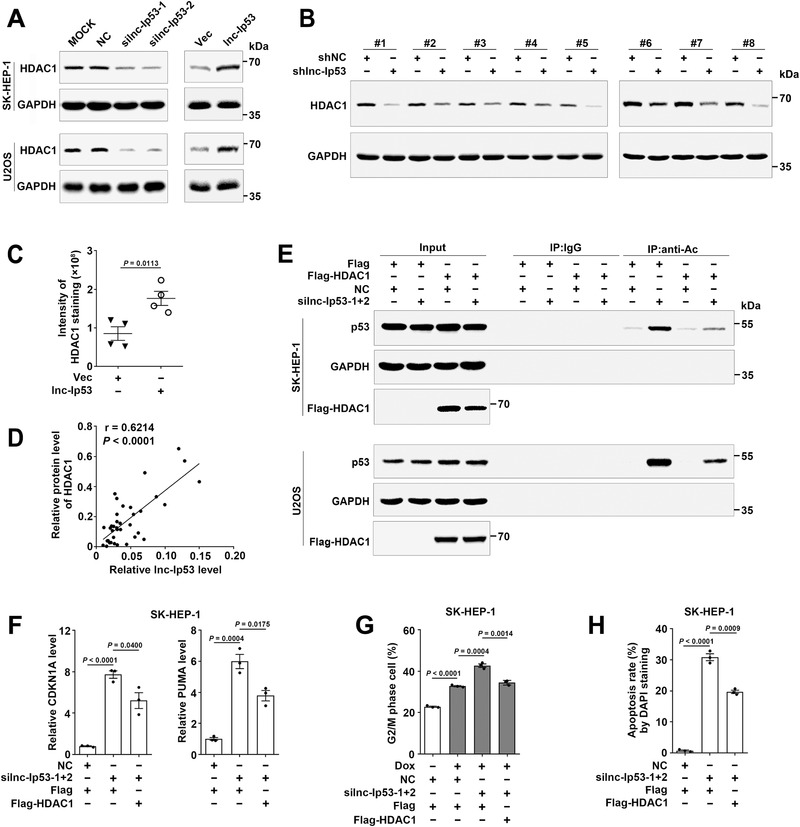
Lnc‐Ip53 inhibits p53 acetylation/activity by increasing HDAC1 level. A) The HDAC1 level was reduced by silnc‐Ip53 (left) and increased by overexpressing lnc‐Ip53 (right). Cells were transfected with the indicated RNA duplexes for 48 h (left) or infected with lentiviruses for 96 h (right) before immunoblotting. MOCK, cells exposed to Lipofectamine RNAiMAX but not RNA duplexes. B) The xenografts with lnc‐Ip53 silencing displayed reduced HDAC1 level. *n*  =  8 mice per group from Figure 4E. C) The xenografts with lnc‐Ip53 overexpression showed increased HDAC1 level. *n*  =  4 mice per group from Figure 4H. D) Spearman′s correlation coefficient analysis revealed a positive association between HDAC1 and lnc‐Ip53 levels in human samples. The RNA levels of lnc‐Ip53 in 19 paired T and N tissues are from Figure 4A and the protein levels of HDAC1 are from Figure S4B in Supporting Information. E) HDAC1 overexpression abrogated silnc‐Ip53‐induced p53 acetylation. Cells expressing Flag or Flag‐HDAC1 were transfected with the indicated RNA duplexes for 42 h, then incubated with 5 µM SAHA for 6 h, followed by IP using IgG or antibody against acetyl lysines (anti‐Ac) and then immunoblotting for p53. F) HDAC1 overexpression abrogated silnc‐Ip53‐stimulated CDKN1A/PUMA expression. Cells expressing Flag or Flag‐HDAC1 were transfected with the indicated RNA duplexes for 52 h before qPCR. G,H) HDAC1 overexpression attenuated silnc‐Ip53‐induced G) G2/M arrest and H) apoptosis. Cells expressing Flag or Flag‐HDAC1 were G) transfected with the indicated RNA duplexes for 39 h and then incubated with 0.1 µM Dox for 9 h before cell cycle analysis, or H) transfected with the indicated RNA duplexes for 72 h before DAPI staining. HDAC1 was analyzed by immunoblotting (A,B,D) or IHC (C). + or −, cells with (+) or without (−) the indicated treatment. Data are shown as mean ± SEM of examined samples (C) or three independent experiments (F–H); *p*‐values were determined by unpaired Student′s *t*‐test; NS, not significant.

Next, the impact of lnc‐Ip53 on the key regulators of p53 acetylation, including acetyltransferases p300/CBP, deacetylases HDAC1/2/3 and SIRT1, was evaluated. We found that silnc‐Ip53 decreased the protein but not mRNA level of HDAC1, and did not affect the levels of other regulators (**Figure** **5**A; Figure S5A,B, Supporting Information). Lnc‐Ip53 overexpression enhanced HDAC1 expression (Figure 5A). Consistently, the protein level of HDAC1 was reduced in lnc‐Ip53‐silencing xenografts (Figure 5B) but enhanced in lnc‐Ip53‐overexpressing xenografts (Figure 5C; Figure S5C, Supporting Information). HDAC1 protein was upregulated in human HCC tissues (Figure S4B and S5D, Supporting Information) and positively correlated with lnc‐Ip53 level (Figure 5D). Furthermore, HDAC1 overexpression attenuated the silnc‐Ip53‐promoted p53 acetylation, CDKN1A/PUMA expression, as well as G2/M arrest and apoptosis (Figure 5E–H; Figure S5E–G, Supporting Information). Consistent with the outcome of silnc‐Ip53, inhibition of HDAC1 by siRNA or SAHA enhanced the levels of acetylated p53 and CDKN1A/PUMA (Figure S5H–J, Supporting Information). Together with the above observations (Figure 4H–J), we suggest that lnc‐Ip53 may inhibit p53 acetylation by enhancing HDAC1 expression.

**Figure 6:**
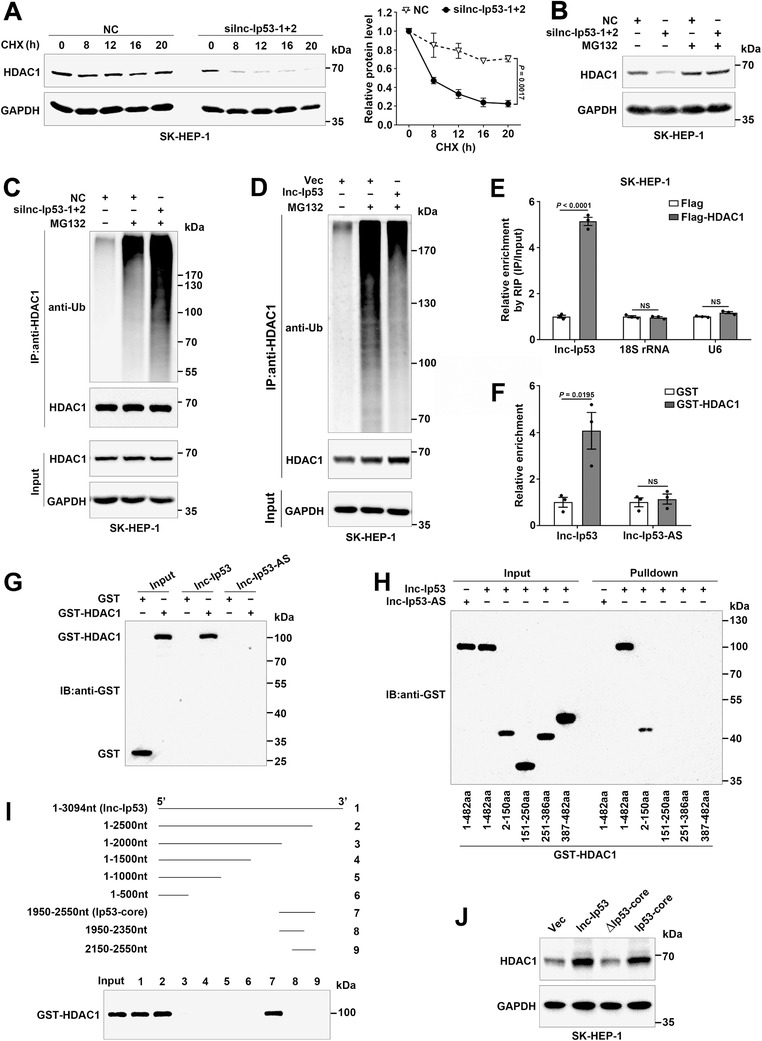
Lnc‐Ip53 interacts with HDAC1 and impedes ubiquitin‐dependent degradation of HDAC1. A) silnc‐Ip53 decreased the stability of HDAC1 protein. Cells were transfected with the indicated RNA duplexes for 32 h, then incubated with 50 µg mL^−1^ cycloheximide (CHX) before immunoblotting. Representative image (left) and quantified level of HDAC1 (right) are shown. B) The proteasome inhibitor MG132 rescued silnc‐Ip53‐induced HDAC1 reduction. Cells were transfected with the indicated RNA duplexes for 36 h, then incubated with 20 µM MG132 for 12 h before immunoblotting. C,D) The ubiquitylated‐HDAC1 level was C) increased by silnc‐Ip53 but D) decreased by lnc‐Ip53 overexpression. Cells were C) transfected with the indicated RNA duplexes for 42 h and then incubated with 20 µM MG132 for 6 h, or D) cells overexpressing lnc‐Ip53 or vector control (Vec) were incubated with 20 µM MG132 for 12 h, followed by IP using anti‐HDAC1 and immunoblotting by ubiquitin antibody (anti‐Ub). E) Lnc‐Ip53 interacted with HDAC1 in vivo. Cells expressing Flag or Flag‐HDAC1 were subjected to RIP using anti‐Flag affinity gel, followed by qPCR for lnc‐Ip53 and negative control genes (18S rRNA, U6). F) GST pulldown assays verified the direct interaction between HDAC1 and lnc‐Ip53 in vitro. The purified GST or GST‐HDAC1 proteins were incubated with lnc‐Ip53 or its antisense RNA (lnc‐Ip53‐AS), followed by GST resin‐precipitation and RNA detection by qPCR. G–I) RNA pulldown assays revealed the direct interaction between the 1950‐2550‐nt region of lnc‐Ip53 (Ip53‐core) and the 2‐150‐aa domain of HDAC1. Biotin‐labeled RNA was incubated with purified GST or GST‐tagged proteins, followed by biotin–streptavidin pulldown and protein detection by immunoblotting (IB) using anti‐GST antibody. In (H), the indicated GST‐tagged HDAC1 fragments were incubated with full‐length lnc‐Ip53 or lnc‐Ip53‐AS. In (I), the indicated lnc‐Ip53 fragments were incubated with full‐length HDAC1. J) The role of lnc‐Ip53 in increasing HDAC1 level was attenuated when Ip53‐core was deleted. Cells overexpressing vector control (Vec) or the indicated lnc‐Ip53 fragments were subjected to immunoblotting. + or −, cells with (+) or without (−) the indicated treatment. Data are shown as mean ± SEM of three independent experiments; *p*‐values were determined by two‐way ANOVA (A) or unpaired Student′s *t*‐test (E,F); NS, not significant.

To explore how lnc‐Ip53 upregulated HDAC1, we first conducted cycloheximide (CHX) chase assay and revealed that silnc‐Ip53 significantly decreased the stability of HDAC1 (**Figure** **6**A), and this effect was abolished by proteasome inhibitor MG132 (Figure 6B). Moreover, the level of ubiquitylated HDAC1 was increased by silnc‐Ip53 (Figure 6C) but decreased by lnc‐Ip53 overexpression (Figure 6D). Both lncPro^[^ ^14^ ^]^ and RPiRLS^[^ ^15^ ^]^ algorithms predicted potential interaction between lnc‐Ip53 and HDAC1, which was then verified experimentally. RNA immunoprecipitation (RIP) assay disclosed that compared with Flag‐control group, the Flag‐HDAC1‐precipitated complex contained more lnc‐Ip53 but similar amount of negative controls, like 18S rRNA or U6 RNA (Figure 6E; Figure S6A, Supporting Information). GST pulldown assays showed that GST‐HDAC1 could precipitate lnc‐Ip53, but not its antisense RNA (lnc‐Ip53‐AS, negative control; Figure 6F; Figure S6B, Supporting Information). Consistently, RNA pulldown assays revealed that GST‐HDAC1 was pulled down by lnc‐Ip53 but not lnc‐Ip53‐AS (Figure 6G). These findings confirmed the interaction of lnc‐Ip53 with HDAC1. Subsequent analysis revealed that only the HDAC1 segments containing the 2‐150‐aa were pulled down by lnc‐Ip53 (Figure 6H), and only the lnc‐Ip53 fragments containing the 1950‐2550‐nt could pull down GST‐HDAC1 (Figure 6I), indicating 1950‐2550‐nt as the core sequence (designated as Ip53‐core) for binding of lnc‐Ip53 to HDAC1. Expectedly, the role of lnc‐Ip53 in increasing HDAC1 level was abrogated when Ip53‐core was deleted, whereas overexpression of Ip53‐core increased the HDAC1 level, which mimicked the effect of full‐length lnc‐Ip53 (Figure 6J). Collectively, lnc‐Ip53 may inhibit ubiquitin‐degradation of HDAC1 protein by directly interacting with HDAC1, which consequently promotes p53 deacetylation.

### 2.5 Lnc‐Ip53 Associates with p300 and Attenuates Its Activity to Acetylate p53

**Figure 7:**
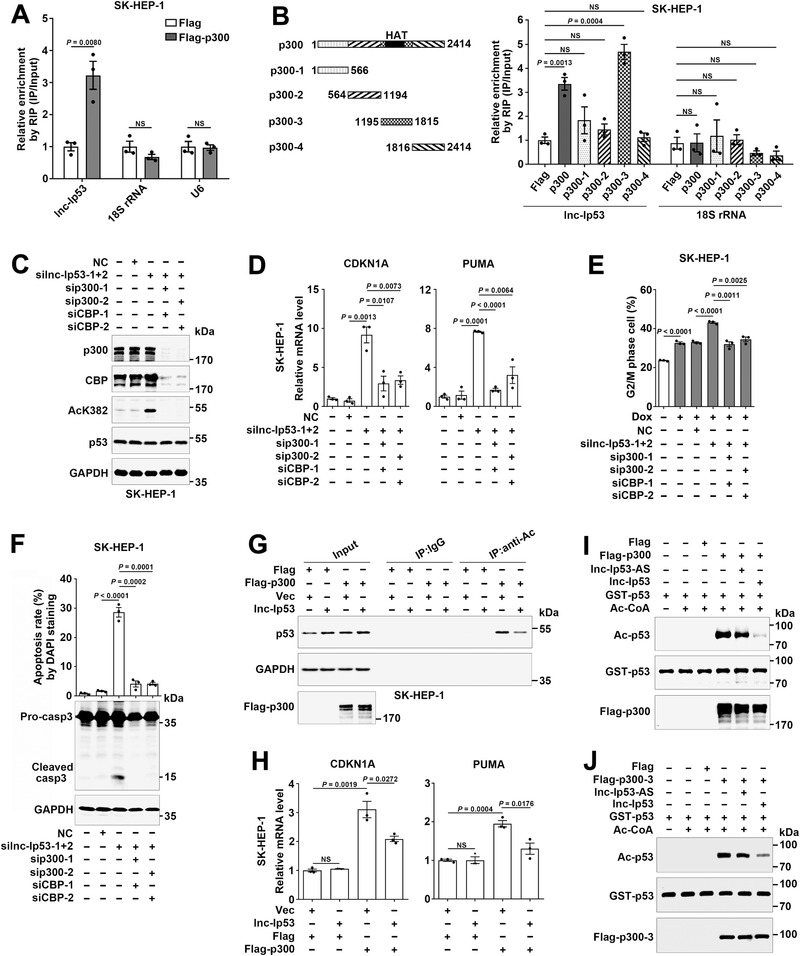
Lnc‐Ip53 associates with p300 and attenuates its activity to acetylate p53. A,B) Lnc‐Ip53 interacted with the 1195‐1815‐aa region of p300 in vivo. Cells expressing Flag or Flag‐tagged p300 fragments were subjected to RIP using anti‐Flag affinity gel, followed by qPCR for lnc‐Ip53 and negative control genes (18S rRNA, U6). C) Silencing p300/CBP (sip300/CBP) attenuated silnc‐Ip53‐stimulated p53 acetylation. Cells were transfected with the indicated RNA duplexes for 42 h, then incubated with 5 µM SAHA for 6 h before immunoblotting for K382‐acetylated p53. D–F) sip300/CBP abrogated the stimulatory effect of silnc‐Ip53 on D) CDKN1A/PUMA expression, E) G2/M arrest, and F) apoptosis. Cells were transfected with the indicated RNA duplexes for 52 h before qPCR (D), or for 39 h, and then incubated with 0.1 µM Dox for 9 h before cell cycle analysis (E), or transfected with the indicated RNA duplexes for 72 h before DAPI staining or 68 h before immunoblotting (F). G) Lnc‐Ip53 attenuated p300‐promoted p53 acetylation. Cells were incubated with 5 µM SAHA for 6 h, followed by IP using IgG or antibody against acetyl lysines (anti‐Ac) and then immunoblotting for p53. H) Lnc‐Ip53 attenuated p300‐induced CDKN1A/PUMA expression. For (G,H), cells infected with control lentiviruses (Vec) or lentiviruses expressing the indicated molecules were used. I,J) The in vitro acetylation assays revealed that lnc‐Ip53 impaired p300 or p300‐3‐mediated p53 acetylation. The purified Flag, I) Flag‐p300 or J) Flag‐p300‐3 proteins were preincubated with lnc‐Ip53 or lnc‐Ip53‐AS, followed by addition of recombinant GST‐p53 and acetyl coenzyme A (Ac‐CoA). The level of acetylated‐p53 was analyzed by immunoblotting using antibody against acetyl lysines (anti‐Ac). + or −, cells with (+) or without (−) the indicated treatment. Data are shown as mean ± SEM of three independent experiments; *p*‐values were determined by unpaired Student′s *t*‐test; NS, not significant.

Both lncPro and RPiRLS predicted that compared with other acetyltransferases, p300/CBP possessed the highest interaction strength with lnc‐Ip53 (Figure S7A, Supporting Information). As p300 and CBP have high sequence and function homology,^[^ ^16^ ^]^ RIP assays were therefore performed using cells expressing Flag‐tagged full‐length p300 or its truncated fragments. Compared with Flag control complex, the Flag‐p300‐precipitated complex contained more lnc‐Ip53 (**Figure** **7**A; Figure S7B, Supporting Information). Moreover, lnc‐Ip53 was also enriched in the precipitates of Flag‐p300‐3, which contains the p53‐interacting domain and HAT domain,^[^ ^3^, ^17^ ^]^ but no lnc‐Ip53 enrichment was observed in the precipitates of other p300 fragments (Figure 7B; Figure S7C, Supporting Information), suggesting the importance of the 1195‐1815‐aa region for the interaction of p300 with lnc‐Ip53. We further investigated whether lnc‐Ip53 regulated p53 acetylation via p300/CBP. Silencing p300/CBP (sip300/CBP) but not MOZ abrogated the silnc‐Ip53‐stimulated p53 acetylation (Figure 7C; Figure S7D,E, Supporting Information) and CDKN1A/PUMA expression (Figure 7D; Figure S7F, Supporting Information). Moreover, sip300/CBP abrogated the silnc‐Ip53‐induced G2/M arrest and apoptosis (Figure 7E,F; Figure S7G,H, Supporting Information). Consistently, lnc‐Ip53 overexpression impaired the p300‐induced p53 acetylation (Figure 7G) and CDKN1A/PUMA upregulation (Figure 7H). Subsequent in vitro acetylation assays revealed that lnc‐Ip53 but not lnc‐Ip53‐AS inhibited the ability of full‐length p300 (Figure 7I) or p300‐3 fragment (Figure 7J) to acetylate p53. These results suggest that lnc‐Ip53 may repress p53 acetylation by interacting with p300 and thereby suppressing p300 activity.

**Figure 8:**
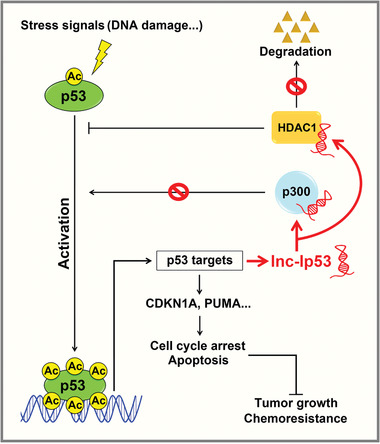
Model of the p53/lnc‐Ip53 feedback loop and its role in tumor growth and chemoresistance.

Taken together, we disclose a novel p53/lnc‐Ip53 negative feedback loop, in which p53 is activated by various stresses, then binds to the lnc‐Ip53 promoter and transactivates lnc‐Ip53 expression, whereas lnc‐Ip53 directly interacts with HDAC1 to prevent its degradation and associates with p300 to attenuate its activity, leading to abrogation of p53 acetylation/activity, which consequently promotes tumor development and chemoresistance (**Figure** **8**).

## 3 Discussion

p53 is a critical tumor suppressor that responds to diverse stresses by orchestrating specific cellular processes, including cell cycle arrest and apoptosis. Acetylation is a vital mechanism to regulate the activity of p53,^[^ ^2^ ^]^ and lncRNAs are emerging as important regulators for protein modifications and activities.^[^ ^7^ ^]^ Nevertheless, the roles of lncRNAs, especially p53‐responsive lncRNAs, in p53 acetylation and their biological significance remain unexplored. We reveal that lncRNA lnc‐Ip53 is transactivated by p53 and in turn represses p53 acetylation and subsequent p53 activation by stabilizing HDAC1 protein and inhibiting p300 activity, thereby forming a negative feedback loop. Abnormal upregulation of lnc‐Ip53, which is detected in different types of cancers, promotes tumor growth and chemoresistance by inhibiting p53 acetylation.

Acetylation is indispensable for p53 activation during stress responses. Enhanced acetylation increases the transcriptional activity of p53, whereas total p53 level is uncoupled from p53 activity.^[^ ^18^, ^19^ ^]^ Loss of acetylation at all eight lysines completely abolishes the p53‐mediated cell cycle arrest and apoptosis.^[^ ^5^ ^]^ Moreover, the p53^KQ/KQ^ knock‐in mouse, which expresses an acetylation‐mimicking mutant p53 with lysine‐to‐glutamine (KQ) substitutions, exhibits a p53 hyperactive phenotype in multiple tissues, confirming the significance of acetylation for p53 activation in vivo.^[^ ^6^ ^]^ Thus, inhibition of p53 acetylation may represent a rapid and effective mechanism to inactivate p53.

It has been reported that p53 can be acetylated by p300/CBP, and deacetylated by HDAC1.^[^ ^2^ ^]^ p300 and CBP share an overall 91% homology in their HAT domains and show similar function.^[^ ^16^ ^]^ Recent reports show that lncRNAs, such as CASC9 and SATB2‐AS1, may interact with p300/CBP and promote acetylation of histone 3, resulting in enhanced gene expression.^[^ ^20^, ^21^, ^22^, ^23^ ^]^ Nevertheless, it is still unknown whether lncRNAs may affect p53 acetylation by interacting with p300 or HDAC1. Here we disclosed that lnc‐Ip53 bound to p300 and decreased p300 activity, thus impaired p53 acetylation. The 1195‐1815‐aa region of p300 (p300‐3) contains the cysteine‐histidine‐rich region 3 (CH3) and HAT domains, which are required for p300 to bind to p53 and to exert its acetyltransferase function, respectively.^[^ ^3^, ^17^ ^]^ Considering that lnc‐Ip53 can interact with p300‐3 and inhibit p300‐3‐mediated acetylation of p53, we therefore speculate that lnc‐Ip53 may compete with p53 for binding to p300, and consequently inhibit p300‐mediated p53 acetylation.

The HDAC family members including HDAC1 are overexpressed in multiple cancer types and several HDAC inhibitors have been approved for cancer treatment.^[^ ^24^ ^]^ HDAC inhibitors, including trichostatin A (TSA), valproic acid (VPA), sodium butyrate, and SAHA, can upregulate acetylated p53 and induce growth arrest and apoptosis,^[^ ^3^, ^25^, ^26^, ^27^ ^]^ suggesting that HDAC‐mediated deacetylation acts as an important mechanism for p53 inactivation and tumor growth. The protein level of HDAC1 is modulated by ubiquitination‐dependent degradation^[^ ^28^, ^29^ ^]^ and microRNA.^[^ ^30^, ^31^ ^]^ However, the lncRNA that regulates HDAC1 expression is not identified yet. We disclosed that lnc‐Ip53 directly bound to the 2‐150‐aa region of HDAC1 and stabilized HDAC1 protein, leading to accumulation of HDAC1 protein and subsequent inhibition of p53 acetylation and abrogation of p53 activity. Given that the 2‐150‐aa region of HDAC1 contains the lysine residue 74 (K74), whose mutation hinders HDAC1 ubiquitination,^[^ ^29^ ^]^ we therefore suppose that binding of lnc‐Ip53 may mask the K74 site of HDAC1 and thereby prevent the ubiquitination and degradation of HDAC1.

Loss of p53 function contributes to tumor growth and chemoresistance.^[^ ^1^ ^]^ A few lncRNAs have been shown to regulate p53 function. For instance, lncRNAs P53RRA and PSTAR are downregulated in tumor tissues. P53RRA interacts with GTPase activating protein (SH3 domain) binding protein 1 (G3BP1) and thus releases p53 from the G3BP1 complex, which promotes p53 nuclear translocation, cell cycle arrest, apoptosis and ferroptosis; enhanced P53RRA expression represses tumor growth.^[^ ^32^ ^]^ PSTAR interacts with heterogeneous nuclear ribonucleoprotein K (hnRNP K) to enhance its SUMOylation, thus promotes the binding of hnRNP K to p53 and stabilizes p53 protein; silencing PSTAR inhibits cell cycle arrest, promotes HCC cells proliferation and tumorigenesis.^[^ ^33^ ^]^ Lnc‐HUR1, which is upregulated by hepatitis B virus, binds to p53 and inhibits its transcriptional activity; enhanced lnc‐HUR1 expression promotes tumor growth and DEN‐induced HCC.^[^ ^34^ ^]^ Nevertheless, it remains unknown whether lncRNA may regulate tumor growth and/or chemoresistance by modulating p53 acetylation. Here, we revealed that lnc‐Ip53 was upregulated in different cancer types and silencing lnc‐Ip53 suppressed the growth of mouse xenografts with wild‐type p53, but not those with acetylation‐resistant p53. Consistently, lnc‐Ip53 overexpression promoted xenograft growth and chemoresistance, which was attenuated by SAHA that promoted p53 acetylation. Further analyses of acetylated p53 and its target genes in mouse xenografts confirmed that lnc‐Ip53 inhibited p53 acetylation/activity in vivo. Moreover, increased level of lnc‐Ip53 was associated with decreased level of acetylated p53 in human HCC and was correlated with poorer survival of HCC with wild‐type p53. Our results suggest that lnc‐Ip53 may promote tumor growth and chemoresistance by repressing p53 acetylation.

As a central hub of the cellular stress response, p53 signaling is amplified or terminated by positive or negative feedback loops, as exemplified by p53‐MDM2 negative feedback circuit. Deregulation of p53‐MDM2 loop resulting from abnormal enhanced MDM2 activity is observed in some cancer cells.^[^ ^35^ ^]^ Though a few lncRNAs have been shown to form feedback loops with p53,^[^ ^9^, ^11^, ^12^ ^]^ only p53‐PURPL regulatory loop was reported to be deregulated in cancer cells. PURPL, which is transactivated by p53, reduces the level of p53 protein by preventing MYBBP1A‐mediated p53 stabilization, and overexpression of PURPL promotes growth of colorectal cancer.^[^ ^12^ ^]^ Here, we characterize a novel p53‐lnc‐Ip53 negative feedback circuit and elucidate its biological significance in cell cycle progression and apoptosis, as well as in tumor development and chemoresistance.

To our knowledge, this is the first report concerning lnc‐Ip53. The findings disclose the regulatory mechanisms of a new lncRNA in the function of p300/CBP and HDAC1 and the acetylation/activity of p53, identify a novel p53/lnc‐Ip53 feedback loop, and implicate lnc‐Ip53 as a potential target for anticancer therapy.

## 4 Experimental Section

##### Reagents

Doxorubicin (Dox, HY‐15142; MedChem Express, Monmouth Junction, NJ, USA), etoposide (HY‐13629; MedChem Express), nutlin‐3a (SC4368; Beyotime, Shanghai, China), MG132 (S1748; Beyotime), cycloheximide (CHX, 2112S; CST, Beverly, MA, USA), suberoylanilide hydroxamic acid (SAHA, S1047; Selleck Chemicals, Houston, TX, USA) were used. PBS was used as vehicle control for Dox, and DMSO as control for etoposide, nutlin‐3a, SAHA, CHX, and MG132.

##### Human Tissues

Human HCC and adjacent non‐tumor liver tissues were collected from patients who underwent HCC resection and confirmed histologically at Sun Yat‐sen University Cancer Center. No local or systemic treatment had been conducted before surgery. Tissues were immediately snap‐frozen in liquid nitrogen until use. Informed consent was obtained from each patient, and the protocol was approved by the Institutional Research Ethics Committee at Sun Yat‐sen University Cancer Center.

##### RNA Oligos

The siRNAs targeting *Homo sapiens lnc‐Ip53* (GeneBank accession No. NR_037177.1), *p53* (NM_000546.5), *p300* (NM_001429.3), *CBP* (NM_004380.2), *MOZ* (NM_006766.4), and *HDAC1* (NM_004964.2) transcripts are designated as silnc‐Ip53, sip53, sip300, siCBP, siMOZ, and siHDAC1, respectively, and were purchased from GenePharma (Shanghai, China). The negative control (NC) RNA duplex for siRNA is nonhomologous to any human genome sequences. The sequences of oligos are listed in Table S2, Supporting Information.

##### Rapid Amplification of cDNA Ends

The 5′‐end and 3′‐end of the lnc‐Ip53 transcript were characterized using 5′‐full rapid amplification of cDNA ends (RACE Kit; TaKaRa, Kyoto, Japan) and 3′RACE System (Invitrogen, Carlsbad, CA, USA), respectively. See also Supporting Information.

##### Vector Construction

Lentivirus expression vectors pCDH‐lnc‐Ip53, pCDH‐Ip53‐core, pCDH‐∆Ip53‐core, pCDH‐p53wt, pCDH‐p53mut, pCDH‐p53/8KR, pCDH‐Flag(N), pCDH‐Flag‐HDAC1, pCDH‐Flag‐p300, pCDH‐Flag‐p300‐1, pCDH‐Flag‐p300‐2, pCDH‐Flag‐p300‐3, pCDH‐Flag‐p300‐4, pCDH‐U6‐shNC, pCDH‐U6‐shlnc‐Ip53, and pCDH‐U6‐shp53, were generated using pCDH‐CMV‐MCS‐EF1‐copGFP (System Biosciences, Palo Alto, CA, USA), which contains a copGFP (copepod green fluorescent protein) expression cassette. GST‐fusion‐protein expression vectors pGEX‐p53, pGEX‐HDAC1, pGEX‐HDAC1/2‐150aa, pGEX‐HDAC1/151‐250aa, pGEX‐HDAC1/251‐386aa, pGEX‐HDAC1/387‐482aa, were generated using pGEX‐6p‐1 (GE Healthcare Bio‐Sciences, Pittsburgh, PA, USA). Luciferase reporter vectors p‐(−1.5/+0.1*k*) and p‐∆p53RE were produced using pGL3‐basic vector (Promega, Madison, WI, USA); p‐p53RE‐wt and p‐p53RE‐mut were generated using pGL3‐promoter vector (Promega). See also Supporting Information.

##### Cell Transfection

RNA duplex at a final concentration of 10 nm and plasmid DNA were transfected using Lipofectamine RNAiMAX and Lipofectamine 2000 (Invitrogen), respectively.

##### Lentivirus Production and Infection

To produce lentiviruses, human embryonic kidney cells expressing SV40 large T antigen (HEK293T) were co‐transfected with lentivirus expression vector and packaging vectors (Lenti‐X HTX Packaging Mix; Clontech, Palo Alto, CA, USA). The lentiviral supernatant was harvested and used to infect target cells. See also Supporting Information.

##### Cell Lines

Human tumor cell lines derived from hepatoma (HepG2, SK‐HEP‐1), osteosarcoma (U2OS), cervical carcinoma (Hela), colorectal carcinoma (HCT116), and HEK293T were used. The stable cell lines SK‐lnc‐Ip53, SK‐∆Ip53‐core, SK‐Ip53‐core, U2OS‐lnc‐Ip53, SK‐Vec, U2OS‐Vec; SK‐Flag‐HDAC1, SK‐Flag‐p300, SK‐Flag‐p300‐1, SK‐Flag‐p300‐2, SK‐Flag‐p300‐3, SK‐Flag‐p300‐4, SK‐Flag; SK‐shlnc‐Ip53, SK‐shNC; SK‐KD‐shlnc‐Ip53‐wt, SK‐KD‐shNC‐wt, SK‐KD‐shlnc‐Ip53‐8KR, SK‐KD‐shNC‐8KR were established by infecting cells with lentiviruses that expressed the target sequence. See also Supporting Information.

##### Analysis of Gene Expression

The gene levels were detected by real‐time quantitative PCR (qPCR), immunoblotting (IB), immunohistochemistry (IHC), and northern blotting. See also Supporting Information.

##### Verification of Protein‐Coding Potential

The protein‐coding potential of lncRNAs was predicted by CPC (http://cpc.cbi.pku.edu.cn/) and CPAT (http://lilab.research.bcm.edu/cpat/index.php) and then experimentally verified. See also Supporting Information.

##### Luciferase Reporter Assay

Luciferase activity was measured using dual‐luciferase reporter assay system (Promega). *Renilla* luciferase expressed by pRL‐TK (Promega) was used as internal control to correct the differences in both transfection and harvest efficiencies. See also Supporting Information.

##### Isolation of Subcellular Fraction

The cytoplasmic and nuclear extracts were isolated using NE‐PER Nuclear and Cytoplasmic Extraction Reagents (Thermo Scientific, Waltham, MA, USA) and validated by immunoblotting using GAPDH and lamin B2 as controls for cytoplasmic and nuclear extracts, respectively.

##### EMSA and Antibody‐Supershift Assay

EMSA was performed using LightShift Chemiluminescent EMSA kit (Thermo Scientific). For competition assay, unlabeled p53 consensus binding oligonucleotides were co‐incubated with nuclear extracts and labeled probe. For antibody‐supershift assay, nuclear extracts were preincubated with anti‐p53 or isotype‐matched IgG. See also Supporting Information.

##### ChIP Assay

ChIP assay was used to investigate the interaction between protein and gene promoter. Cells (3–5 × 10^6^) were cross‐linked with 1% formaldehyde at room temperature (RT) for 10 min, incubated with 125 mm glycine at RT for another 3 min and washed twice with ice‐cold PBS. The cells were collected with 2 mL of DTT solution (100 mm Tris‐HCl at pH 9.5, 10 mm DTT) and incubated at RT for 10 min followed by centrifugation at 5000 *g* and 4 °C for 5 min. The cell pellets were resuspended in 150 µL of SDS lysis buffer (50 mm Tris‐HCl at pH 8.0, 2 mm EDTA and 1% SDS) supplemented with protease inhibitor cocktail (Bimake, Houston, TX, USA), sonicated at 4 °C for 4 min (30 s on and 30 s off) on Bioruptor (Diogenode, Liege, Belgium) under the high‐power model, then centrifuged at 13 000 *g* and 4 °C for 10 min. The supernatants were mixed with twofold volume of dilution buffer (20 mm Tris‐HCl at pH 8.0, 200 mm NaCl, 2 mm EDTA, 0.1% sodium deoxycholate, 1% Triton X‐100, and protease inhibitor cocktail). The cross‐linked chromatin complexes in the supernatants were immunoprecipitated at 4 °C overnight with 2 µg of antibodies against p53 (sc‐126; Santa Cruz Biotechnology, Dallas, TX, USA), K382‐acetylated p53 (ab75754; Abcam, Cambridge, MA, USA), isotype‐matched mouse control IgG (A7028; Beyotime) or rabbit control IgG (A7016; Beyotime), then collected by incubation with 10 µL of protein A/G magnetic beads (Bimake) at 4 °C for 2 h. The beads were washed five times with ice‐cold IP lysis buffer (25 mm Tris‐HCl at pH 7.4, 150 mm NaCl, 1 mm EDTA, 1% NP‐40, 5% glycerol and protease inhibitor cocktail), then eluted in 400 µL of elution buffer (0.1 m NaHCO_3_, 1% SDS) and rotated at RT for 1 h, followed by addition of 8 µL of 0.5 m EDTA and 16 µL of 1 m Tris‐HCl at pH 6.5 and 60 µg of proteinase K (TaKaRa). The eluted chromatin complexes were incubated at 65 °C overnight to reverse DNA‐protein crosslink. The DNA was then purified and analyzed by qPCR or semi‐quantitative PCR using specific primers listed in Table S2, Supporting Information. The promoters of CDKN1A and GAPDH were used as positive and negative controls, respectively.

##### Immunoprecipitation Assay

IP assay was performed as previously described^[^ ^36^ ^]^ with modifications. Briefly, cells (2 × 10^6^) were resuspended in 250 µL of NP‐40 lysis buffer (20 mm Tris‐HCl at pH 7.4, 150 mm NaCl, 1 mm EDTA, 1% NP‐40, 10% glycerol and protease inhibitor cocktail) and incubated at 4 °C for 30 min, followed by centrifugation at 13 000 *g* and 4 °C for 10 min. The supernatants were incubated with 2 µg of antibodies against acetyl lysines (ab21623; Abcam), HDAC1 (sc‐81598; Santa Cruz Biotechnology) or isotype‐matched IgG at 4 °C for 6 h. Then the immunoprecipitated complexes were collected by incubation with 10 µL of protein A/G magnetic beads (Bimake) at 4 °C for 2 h. The beads were washed three times with washing buffer (50 mm Tris‐HCl at pH 7.4, 150 mm NaCl, 0.1% Triton X‐100 and protease inhibitor cocktail), eluted in 1 × SDS buffer (50 mm Tris‐HCl at pH 6.8, 2% SDS, 0.01% bromophenol blue, 10% glycerol, 1% 2‐mercaptoethanol and protease inhibitor cocktail) and boiled for 10 min. The proteins were retrieved and subjected to immunoblotting.

##### RIP Assay

RIP assay was used to explore the interaction between protein and RNA and performed as previously described^[^ ^37^ ^]^ with modifications. Briefly, cells (2 × 10^7^) expressing Flag or Flag‐tagged proteins were cross‐linked, resuspended in 0.5 mL of ice‐cold RIPA lysis buffer (50 mm Tris‐HCl at pH 8.0, 150 mm KCl, 5 mm EDTA, 0.1% SDS, 1% Triton X‐100, 0.5% sodium deoxycholate, 0.5 mm DTT, 500 U mL^−1^ RNase inhibitor [Promega] and protease inhibitor cocktail), sonicated at 4 °C for 4 min (30 s on and 30 s off) on Bioruptor under the low‐power model, then centrifuged at 13 000 *g* and 4 °C for 10 min. The supernatants were mixed with equal volume of RIP binding/wash buffer (25 mm Tris‐HCl at pH 7.5, 150 mm KCl, 5 mm EDTA, 0.5% NP‐40, 0.5 mm DTT, 500 U mL^−1^ RNase inhibitor and protease inhibitor cocktail). The cross‐linked RNA‐protein complexes in the supernatants were immunoprecipitated by incubation with 20 µL of anti‐Flag affinity gel (Bimake) at 4 °C for 4 h. The affinity gel was washed three times with RIP binding/washing buffer, then resuspended in 50 µL of IP lysis buffer (25 mm Tris‐HCl at pH 7.4, 150 mm NaCl, 1 mm EDTA, 1% NP‐40 and 5% glycerol) supplemented with 1 µL of RNase inhibitor and 60 µg of proteinase K, followed by incubation at 55 °C for 1 h and at 70 °C for another 45 min to reverse RNA‐protein crosslink. The RNA was extracted by TRIzol and analyzed by qPCR. The sequences of primers are listed in Table S2, Supporting Information.

##### Purification of GST‐Fusion Proteins

*Escherichia coli* (*E. coli*) BL21 (DE3; TaKaRa) carrying vectors expressing GST or GST‐fusion proteins were grown in LB (Sangon Biotech, Shanghai, China) at 37 °C with shaking until OD_600_ was about 0.6, followed by addition of 0.1 mm IPTG (Sangon Biotech) and incubation at 22 °C for another 8 h. The *E. coli* were resuspended in ice‐cold PBS supplemented with protease inhibitor cocktail and 1 mm DTT, sonicated at 4 °C for 60 min (5 s on and 15 s off) on Bioruptor under the high‐power model, then centrifuged at 15 000 *g* and 4 °C for 30 min. GST‐fusion proteins in the supernatants were purified by GST‐sefinose (TM) resin (Sangon Biotech) according to the manufacturer's instructions. All proteins were concentrated in BC100 buffer (20 mm Tris–HCl at pH 8.0, 0.5 mm EDTA, 100 mm KCl, 20% glycerol, 0.5 mm DTT, 0.5 mm PMSF [Beyotime], and protease inhibitor cocktail) by Amicon Ultra‐15 Centrifugal Filter Devices (UFC901008; Millipore, Billerica, MA) and stored at −80 °C. The protein concentration was measured by BCA Protein Assay Kit (Beyotime).

##### In Vitro Transcription

For GST pulldown, RNA pulldown and in vitro acetylation assays, biotin‐labeled RNAs and unlabeled RNAs were in vitro transcribed from the relevant PCR products using Biotin RNA Labeling Mix (Roche, Mannheim, Germany) and NTP Mix (Invitrogen) together with T7 RNA polymerase (Promega), respectively, then purified by LiCl precipitation. The purified RNAs were incubated in annealing buffer (Beyotime) at 90 °C for 2 min, placed on ice for 2 min and kept at RT for 20 min to form secondary structure. The sequences of primers are listed in Table S2, Supporting Information.

##### GST Pulldown Assay

GST pulldown assays were performed as previously described^[^ ^38^ ^]^ with modifications. To examine in vitro interaction between HDAC1 and lnc‐Ip53, 2 µg of purified GST or GST‐HDAC1 proteins were incubated with 0.5 µg of in vitro transcribed unlabeled RNA in 500 µL of RIP binding/wash buffer (25 mm Tris‐HCl at pH 7.5, 150 mm KCl, 5 mm EDTA, 0.5% NP‐40, 0.5 mm DTT, 500 U mL^−1^ RNase inhibitor and protease inhibitor cocktail) at 4 °C for 2 h, and collected by incubation with 100 µL of BeyoGold GST‐tag purification resin (Beyotime) at 4 °C for 6 h. The resin was washed five times with ice‐cold RIP binding/wash buffer. The RNA in the resin‐precipitates was extracted by TRIzol and analyzed by qPCR. The sequences of primers are listed in Table S2, Supporting Information.

##### RNA Pulldown Assay

To preclear the unspecific proteins, 20 µL of streptavidin MagneSphere paramagnetic particles (Promega) were washed by IP lysis buffer (25 mm Tris‐HCl at pH 7.4, 150 mm NaCl, 1 mm EDTA, 1% NP‐40, 5% glycerol, protease inhibitor cocktail, and 500 U mL^−1^ RNase inhibitor), and the paramagnetic particles were then collected and added to 250 µL of IP lysis buffer that contained 1 µg of purified GST or GST‐fusion proteins, followed by incubation at 4 °C for 1.5 h. The precleared supernatants were incubated subsequently with 0.5 µg of in vitro transcribed biotin‐labeled RNA at 4 °C for 2.5 h and 40 µL of prewashed streptavidin MagneSphere paramagnetic particles at 4 °C for another 2.5 h. The paramagnetic particles were collected, washed four times with high‐salt washing buffer (25 mm Tris‐HCl at pH 7.4, 300 mm NaCl, 1 mm EDTA, 1% NP‐40, 5% glycerol, 100 U mL^−1^ RNase inhibitor and protease inhibitor cocktail), eluted in 1 × SDS buffer (50 mm Tris‐HCl at pH 6.8, 2% SDS, 0.01% bromophenol blue, 10% glycerol, 1% 2‐mercaptoethanol and protease inhibitor cocktail) and boiled for 10 min. The proteins were retrieved from the particle‐precipitates and subjected to immunoblotting.

##### In vitro Acetylation Assay

To obtain Flag, Flag‐p300 or Flag‐p300‐3 proteins, HEK293T cells at 20–30% confluence in a 10‐cm dish were transfected with 10 µg of pCDH‐Flag, pCDH‐Flag‐p300 or pCDH‐Flag‐p300‐3 by calcium phosphate. After 48 h, the cells were lysed in 1 mL of IP lysis buffer (25 mm Tris‐HCl at pH 7.4, 150 mm NaCl, 1 mm EDTA, 1% NP‐40, 5% glycerol and protease inhibitor cocktail) at 4 °C for 30 min, then incubated with 20 µL of anti‐Flag affinity gel at 4 °C for 4 h. The affinity gel was washed three times with high‐salt washing buffer (25 mm Tris‐HCl at pH 7.4, 300 mm NaCl, 1 mm EDTA, 1% NP‐40, 5% glycerol, and protease inhibitor cocktail), resuspended in 60 µL of 2 × HAT buffer (100 mm Tris‐HCl at pH 8.0, 20% glycerol, 2 mm DTT, 0.2 mm EDTA, 20 mm sodium butyrate, 2 mm PMSF, protease inhibitor cocktail) and stored in aliquots at −80 °C.

Protein acetylation assay was then performed as reported^[^ ^39^ ^]^ with modifications. Ten µL of affinity gel‐bound Flag, Flag‐p300 or Flag‐p300‐3 were preincubated with 1 µg of in vitro transcribed unlabeled lnc‐Ip53 or lnc‐Ip53‐AS at RT with shaking (800 r.p.m.) for 30 min, followed by adding 1 µg of purified GST‐p53, 0.5 mm acetyl coenzyme A (Ac‐CoA; A2056; Sigma‐Aldrich, St Louis, MO, USA), 2 × HAT buffer and DEPC‐H_2_O to make up a final volume of 30 µL reaction mixture containing 1 × HAT buffer, then incubated at 30 °C with shaking (800 r.p.m.) for 90 min before immunoblotting.

##### Cell Cycle Analysis

Cells were stained with propidium iodide (PI), then analyzed by fluorescence‐activated cell sorting (FACS; Gallios, Beckman Coulter, Miami, FL, USA). See also Supporting Information.

##### Apoptosis Analysis

Apoptosis was evaluated by 4′‐6′‐diamidino‐2‐phenylindole (DAPI; Sigma‐Aldrich) staining for morphology examination, annexin V staining for FACS analysis and immonublotting for cleaved caspase‐3. See also Supporting Information.

##### Cell Counting Assay

Cells were cultured in a 24‐well plate for 6 days before cell counting by Countstar (ALIT Life Sciences, Shanghai, China). See also Supporting Information.

##### Colony Formation Assay

Cells were maintained in a 6‐well plate for 10 days (SK‐HEP‐1) or 14 days (U2OS). Colonies were stained with crystal violet solution and counted. See also Supporting Information.

##### Mouse Xenograft Models

Male NOD‐Prkdcem26Cd52Il2rgem26Cd22/Nju (NCG) mice at 5 weeks of age were used. For loss‐of‐function study, mice were subcutaneously injected with SK‐shNC or SK‐shlnc‐Ip53 cells (4 × 10^6^) in 100 µL of DMEM/matrigel (1:1 volume; 3432‐005‐01 for matrigel; R&D Systems, Minneapolis, MN, USA). Mice were sacrificed 27 days after implantation.

To validate whether lnc‐Ip53 exerted its function by regulating p53 acetylation in vivo, mice were implanted with SK‐KD‐shNC‐wt, SK‐KD‐shlnc‐Ip53‐wt, SK‐KD‐shNC‐8KR or SK‐KD‐shlnc‐Ip53‐8KR cells (4 × 10^6^) in 100 µL of DMEM/matrigel (1:1 volume). Mice were sacrificed 30 days after implantation.

To evaluate the influence of lnc‐Ip53 on chemoresistance, 10 days after subcutaneous injection with SK‐Vec or SK‐lnc‐Ip53 cells (3 × 10^6^) in 100 µL of DMEM/matrigel (1:1 volume), mice were administered with vehicle(1 × PBS as vehicle control for Dox, iv; corn oil as control for SAHA, ip), Dox (3 mg kg^−1^, iv) or combined SAHA (75 mg kg^−1^, ip) and Dox (3 mg kg^−1^). Dox was administered twice a week for a total of six times and SAHA was administered every day. Mice were sacrificed 30 days after implantation.

Tumor growth was monitored every 3 days and the volume of tumor was measured with electronic digital calipers and calculated with the formula: volume = (length × width^2^)/2. At the end of experiments, tumors were excised, photographed, and weighed. Aliquots of tumor tissues were stored in liquid nitrogen for RNA and protein isolation, and fixed in 10% formalin and embedded in paraffin for IHC.

All mouse experiments were approved by the Institutional Animal Care and Use Committee at Sun Yat‐sen University. All procedures for animal experiments were performed in accordance with the Guide for the Care and Use of Laboratory Animals (NIH publications Nos. 80‐23, revised 1996) and according to the institutional ethical guidelines for animal experiments.

##### Bioinformatics

The potential binding sites of transcription factors on the lnc‐Ip53 promoter were predicated using MAPPER.^[^ ^40^ ^]^ The *p53* mutation status and clinical features of HCC were downloaded from cBioPortal (http://www.cbioportal.org/) and The Cancer Genome Atlas (TCGA; https://portal.gdc.cancer.gov/), respectively. The data of lnc‐Ip53 expression from TCGA cohort were downloaded from TANRIC (https://ibl.mdanderson.org/tanric/_design/basic/main.html).

##### Statistical Analyses

All statistical analyses were performed using GraphPad Prism version 8.0.1 (GraphPad Software Inc., San Diego, CA, USA). Data are shown as mean ± SEM from at least three independent experiments. Unpaired or paired Student′s *t*‐test, or two‐way ANOVA were carried out to compare the differences between two groups. The variances are similar between the groups that are being statistically compared. The statistical tests are justified as appropriate and meet the assumptions of the tests. Survival curves were calculated using Kaplan–Meier method, and *p*‐value was determined by log‐rank test. Recurrence‐free survival was calculated from the date of HCC resection to the time of first recurrence or death. Patients who were lost to follow‐up were treated as censored events. Spearman′s correlation coefficient analysis was applied to examine the correlation between the levels of lnc‐Ip53 and acetylated p53 or HDAC1. A *p*‐value of less than 0.05 was considered as the criterion of statistical significance, and all statistical tests were two‐tailed.

## Conflict of Interest

The authors declare no conflict of interest.

## Author Contributions

L.Z.Z. and J.E.Y. contributed equally to this work. L.Z.Z. and J.E.Y. designed and performed the experiments, analyzed the data, and wrote the manuscript. Y.W.L. and F.T.L. performed the experiments and analyzed the data. Y.F.Y. provided human samples and interpreted clinical data. S.M.Z. supervised and organized the study, designed the experiments, analyzed the data, and wrote the manuscript.

## Supporting information

Supporting Information
