# Supplementary material for: A p53/lnc‐Ip53 Negative Feedback Loop Regulates Tumor Growth and Chemoresistance
Source: Adv Sci (Weinh). 2020 Sep 28;7(21):2001364. doi: 10.1002/advs.202001364 (PMC7610266; doi:10.1002/advs.202001364)
Supplement: Supplementary file 1 — Supporting Information [file ADVS-7-2001364-s001.pdf]

© 2020 Wiley-VCH GmbH

Supporting Information

for *Adv. Sci.*, DOI: 10.1002/advs.202001364

A p53/lnc-Ip53 Negative Feedback Loop Regulates Tumor Growth and Chemoresistance

*Li-Zhen Zhang, Jin-E Yang, Yu-Wei Luo, Feng-Ting Liu, Yun-Fei Yuan and Shi-Mei Zhuang**

Supporting Information

A p53/lnc-Ip53 Negative Feedback Loop

Regulates Tumor Growth and Chemoresistance

Li-Zhen Zhang, Jin-E Yang, Yu-Wei Luo, Feng-Ting Liu, Yun-Fei Yuan and Shi-Mei Zhuang[*]

Inventory of supplementary data

1. Supplementary Experimental Section...…………..…. Page 2 - 10

2. Supplementary figures and legends……….….………Page 11 - 24

3. Supplementary tables…………………….……..……… Page 25 - 28

Supplementary Experimental Section

Rapid amplification of cDNA ends (RACE): The 5′-end of the lnc-Ip53 transcript was characterized using 5′-Full RACE Kit (TaKaRa, Kyoto, Japan). Briefly, total RNA from SK-HEP-1 cells was treated with calf intestinal phosphatase (CIAP) and tobacco acid pyrophosphatase (TAP), and then ligated to a 5′RACE RNA adaptor, followed by reverse-transcription with random primers and nested PCR using gene specific primers (5′RACE-GSP1 and 5′RACE-GSP2) to amplify the 5′-end of the transcript.

The 3′-end of lnc-Ip53 was characterized using the 3′RACE System (Invitrogen, Carlsbad, CA, USA). Total RNA from SK-HEP-1 cells was subjected to reverse-transcription with a 3′RACE-adaptor primer, followed by nested PCR using gene specific primers (3′RACE-GSP1 and 3′RACE-GSP2) and abridged universal amplification primer (AUAP). The PCR products were verified by direct sequencing. The sequences of primers are listed in Table S2, Supporting Information.

Vector construction: The following vectors were used: lentivirus expression vectors, including pCDH-lnc-Ip53, pCDH-Ip53-core, pCDH-ΔIp53-core, pCDH-p53wt, pCDH-p53mut, pCDH-p53/8KR, pCDH-Flag(N), pCDH-Flag-HDAC1, pCDH-Flag-p300, pCDH-Flag-p300-1, pCDH-Flag-p300-2, pCDH-Flag-p300-3, pCDH-Flag-p300-4, were generated using pCDH-CMV-MCS-EF1-copGFP (System Biosciences, Palo Alto, CA, USA), which contains a copGFP (copepod green fluorescent protein) expression cassette. Vectors expressing GST-fusion proteins, including pGEX-p53 and pGEX-HDAC1, pGEX-HDAC1/2-150aa, pGEX-HDAC1/151-250aa, pGEX-HDAC1/251-386aa, pGEX-HDAC1/387-482aa, were generated using pGEX-6p-1 (GE Healthcare Bio-Sciences, Pittsburgh, PA, USA). The details for vector construction were described as follows.

pCDH-lnc-Ip53, pCDH-Ip53-core and pCDH-ΔIp53-core were produced by inserting full-length lnc-Ip53, the 1950-2550-nt of lnc-Ip53 or the mutant lnc-Ip53 with a 1950-2550-nt

deletion into the *Nhe*I/*Eco*RI sites of pCDH-CMV-MCS-EF1-copGFP using ClonExpress® One Step Cloning Kit (Vazyme, Nanjing, China).

To create pCDH-p53wt, the coding sequence of *p53* was inserted into the *Eco*RI/*Bam*HI sites of pCDH-CMV-MCS-EF1-copGFP. pCDH-p53mut, which contained an arginine to histidine substitution at codon 175 of p53, was generated by fusion PCR based on pCDH-p53wt. pCDH-p53/8KR, which had lysine to arginine substitution at the codons 120, 164, 370, 372, 373, 381, 382 and 386 of p53, was generated by fusion PCR based on the pCDH-p53wt.

To construct the plasmids that express target protein containing Flag-tag in the N-terminal, the backbone plasmid pCDH-Flag(N) was produced by inserting 3×FLAG sequence with *Xho*I and *Eco*RV restriction enzyme digestion sites into the *Xba*I/*Eco*RI sites of pCDH-CMV-MCS-EF1-copGFP. pCDH-Flag-HDAC1 and pCDH-Flag-p300 were generated by respectively cloning the coding sequence of *HDAC1* and *p300* into the *Xho*I/*Eco*RI sites of pCDH-Flag(N). The plasmids pCDH-Flag-p300-1, pCDH-Flag-p300-2, pCDH-Flag-p300-3 and pCDH-Flag-p300-4, which respectively expressed the 1-566aa, 564-1194aa, 1195-1815aa and 1816-2414aa domains of p300, were generated by cloning the relevant nucleotides of *p300* coding sequence into the *Xho*I/*Eco*RI sites of pCDH-Flag(N).

To generate pCDH-U6-shNC, pCDH-U6-shlnc-Ip53 and pCDH-U6-shp53 vectors, the complementary oligonucleotides that contained both sense and antisense siRNA sequences (shNC: 5′- TGA ATT AGA TGG CGA TGT T; shlnc-Ip53: 5′-GTT GAA GGC TGG TGC AGT A; shp53: 5′-GGA GGA TTT CAT CTC TTG TAT), the spacer sequence (5′-TTC AAGA GA) and the flanking *Eco*RI and *Bam*HI sites were chemically synthesized, annealed and then inserted into the *Eco*RI/*Bam*HI sites in the pCDH-U6 vector, which was produced based on the pCDH-CMV-MCS-EF1-copGFP vector by replacing the CMV promoter with the U6 promoter.

To construct GST-fusion proteins, the coding sequence of *HDAC1* and *p53* were respectively inserted into the *Bam*HI/*Xho*I sites of pGEX-6p-1 using ClonExpress® One Step

Cloning Kit. The plasmids pGEX-HDAC1/2-150aa, pGEX-HDAC1/151-250aa, pGEX-HDAC1/251-386aa and pGEX-HDAC1/387-482aa were generated by cloning the relevant nucleotides of *HDAC1* coding sequence into the *Bam*HI/*Xho*I sites of pGEX-6p-1.

To construct the promoter reporter p-(−1.5/+0.1k), the −1500 ~ +100 bp sequence (chr6:32861049-32862648) of lnc-Ip53 was cloned into the *Nhe*I/*Hin*dIII sites upstream of the coding sequence of *firefly* luciferase in pGL3-basic vector (Promega, Madison, WI, USA). The plasmid p-Δp53RE, which had a deletion of the predicted p53-responsive element (p53RE), was generated by fusion PCR based on the p-(−1.5/+0.1k).

To verify whether p53 regulated gene transcription by binding to the p53RE in lnc-Ip53, the complementary oligonucleotides that contained both sense and antisense sequences of wild-type or mutant p53RE (wild-type: 5′-GGA GCG CCC AGG GCA TGT CC; mutant: 5′-GGA GCG CCC ATT TAC GTA GG) and the flanking sites of *Nhe*I and *Bgl*II were chemically synthesized, annealed and then inserted into the *Nhe*I/*Bgl*II sites upstream of the SV40 promoter in pGL3-promoter vector (Promega). The generated vectors were designated as p-p53RE-wt and p-p53RE-mut.

All constructs were verified by sequencing. All sequences of oligonucleotide and primers are listed in Table S2, Supporting Information.

Lentivirus production and infection: To produce lentiviruses, human embryonic kidney cell expressing SV40 large T antigen (HEK293T) cells were co-transfected with lentivirus expression vector and packaging vectors (Lenti-X HTX Packaging Mix; Clontech, Palo Alto, CA, USA) by calcium phosphate, then refreshed with culture medium 16 hours after transfection. Thirty-six hours later, the lentiviral supernatant was harvested and stored in aliquots at −80 °C until use. Target cells were infected with lentiviral supernatant supplemented with 8 μg mL^{-1} polybrene (Millipore, Billerica, MA), then subjected to further experiment 96 hours post-infection.

Cell lines: Human tumor cell lines derived from hepatoma (HepG2, SK-HEP-1), osteosarcoma (U2OS), cervical carcinoma (Hela), colorectal carcinoma (HCT116) and HEK293T were cultured in Dulbecco′s modified Eagle′s medium (DMEM; Life Technologies, Gaithersburg, MD, USA) supplemented with 10% fetal bovine serum (FBS; Hyclone, Logan, UT, USA), 100 U mL^{-1} penicillin and 100 μg mL^{-1} streptomycin in a humidified atmosphere of 5% CO_2 at 37 °C.

The stable cell lines were established by infecting SK-HEP-1 or U2OS cells with lentiviruses that expressed the target sequence. These included sublines with stable expression of lnc-Ip53 (SK-lnc-Ip53, U2OS-lnc-Ip53) or truncated lnc-Ip53 (SK-ΔIp53-core and SK-Ip53-core) and their control lines (SK-Vec, U2OS-Vec); sublines with stable expression of Flag-tagged HDAC1 (SK-Flag-HDAC1) or p300 (SK-Flag-p300) or truncated p300 (SK-Flag-p300-1, SK-Flag-p300-2, SK-Flag-p300-3, SK-Flag-p300-4) and their control line (SK-Flag); sublines with stable silencing of lnc-Ip53 (SK-shlnc-Ip53) and its control line (SK-shNC). To establish cell lines used for validating the causative roles of lnc-Ip53 on p53 acetylation *in vivo*, the subline with stable silencing of p53 (SK-KD) was first constructed using SK-HEP-1 cells, and then SK-KD-shlnc-Ip53 with stable silencing of lnc-Ip53 and its control line SK-KD-shNC were generated, followed by infection with lentiviruses that expressed wild-type p53 (SK-KD-shNC-wt, SK-KD-shlnc-Ip53-wt) or expressed acetylation-resistant p53 that had lysine to arginine mutation in eight acetylation sites of p53 (SK-KD-shNC-8KR, SK-KD-shlnc-Ip53-8KR).

Analysis of gene expression: Real-time quantitative PCR (qPCR) was used to detect RNA levels. For mRNA and lncRNA analysis, total RNA was extracted using TRIzol reagent (Invitrogen), then reverse-transcripted with random primers (Sangon Biotech, Shanghai, China) using M-MLV reverse transcriptase (Promega), followed by qPCR on a LightCycler 480 (Roche, Mannheim, Germany) using SYBR Green qPCR Master Mix (Bimake, Houston, TX, USA).

All reactions were performed in duplicate. The cycle threshold (Ct) values differed by less than 0.3 between duplicate wells. The relative expression levels of the target genes were normalized to that of internal control genes, which yielded a $2^{-\Delta Ct}$ value. U6 and GAPDH were used as the internal control genes for the relative expression levels in tissues and cell lines, respectively.

Immunoblotting (IB) was performed to examine the protein levels. The antibodies used included: mouse monoclonal antibodies (mAbs) against p53 (sc-126; Santa Cruz Biotechnology, Dallas, TX, USA), HDAC1 (sc-81598; Santa Cruz Biotechnology), HDAC2 (AH382; Beyotime), HDAC3 (sc-376957; Santa Cruz Biotechnology), Flag (F1804; Sigma-Aldrich, St Louis, MO, USA), GST (RM1005; Beijing Ray Antibody Biotech, Beijing, China); rabbit mAbs against SIRT1 (ab32441; Abcam, Cambridge, MA, USA), K382-acetylated p53 (ab75754; Abcam), ubiquitin (ab134953; Abcam), CBP (7389S; CST), lamin B2 (12255; CST), GAPDH (BM1623; Boster, Wuhan, China); rabbit polyclonal antibodies against caspase-3 (9662S; CST), phospho-p53 (Ser6) (9285T; CST), phospho-p53 (Ser9) (9288S; CST), phospho-p53 (Ser15) (9284T; CST), phospho-p53 (Thr18) (2529T; CST), phospho-p53 (Ser20) (9287T; CST), phospho-p53 (Ser392) (9281T; CST), p300 (YT3508; ImmunoWay, Plano, TX, USA), acetyl lysines (ab21623; Abcam).

Immunohistochemistry (IHC) was used to examine protein levels in xenograft tissues. Formalin-fixed, paraffin-embedded tissues were cut into 5-μm sections, deparaffinized in xylene, rehydrated through graded ethanol and applied to IHC staining using mouse mAb against HDAC1 (sc-81598; Santa Cruz Biotechnology) or rabbit mAb against K382-acetylated p53 (ab75754; Abcam). The intensity of HDAC1 or acetylated p53 staining was calculated based on per unit areas using Aperio Positive Pixel Count v9 Algorithm (Aperio/Leica Microsystems, Vista, CA, USA).

Northern blotting was performed as previously described.[1] Briefly, the total RNA from SK-HEP-1 cells treated with 1μM Dox for 24 hours was isolated using TRIzol and subjected to

northern blotting. Twenty μg of total RNA was loaded in a 1.2% denaturing formaldehyde-agarose gel to separate the RNA transcripts by electrophoresis, then transferred onto Amersham Hybond^{TM}-N^+ membrane (GE Healthcare, Menlo Park, California, USA) by capillary transfer for 8 hours, followed by 120000 μJ cm^{-2} UV cross-linking. The membrane was prehybridized at 42 °C overnight in 2× SSC buffer (Sangon Biotech) with 100 μg mL^{-1} salmon sperm DNA (Sangon Biotech) and then incubated with 20 pM digoxin-labeled oligonucleotide probe at 42 °C overnight, followed by stringent washing and signal detection using anti-digoxigenin AP-conjugate (Roche).

Verification of protein-coding potential: The protein-coding potential of lncRNAs was predicted by CPC (http://cpc.cbi.pku.edu.cn/) and CPAT (http://lilab.research.bcm.edu/cpat/index.php) and then experimently verified. Expression plasmids of the top two predicted open reading frames (ORFs) in lnc-Ip53 gene were constructed, in which the predicted ORFs and its 5′UTR sequence were fused in frame to the N-terminus of GFP (without ATG) and cloned into a pcDNA3.0 (Invitrogen) plasmid. The expression of GFP-fusion proteins was detected by fluorescence microscopy (Nikon, Melville, NY, USA) 48 hours after plasmid transfection. All sequences of primers are listed in Table S2, Supporting Information.

Luciferase reporter assay: Luciferase activity was measured using dual-luciferase reporter assay system (Promega). *Renilla* luciferase expressed by pRL-TK (Promega) was used as internal control to correct the differences in both transfection and harvest efficiencies.

To characterize the lnc-Ip53 promoter, cells in a 48-well plate were co-transfected with 25 ng pRL-TK and 100 ng p-(−1.5/+0.1k) for 48 hours before luciferase activity assay. To investigate the effect of Dox on the lnc-Ip53 promoter, cells were co-transfected with 25 ng pRL-TK, 100 ng p-(−1.5/+0.1k) or p-Δp53RE for 24 hours, followed by incubation with 0.5 μM Dox for another 24 hours. To test the effect of silencing p53 on the lnc-Ip53 promoter, cells

were reversely transfected with 10 nM sip53 or NC duplex for 24 hours, then co-transfected with 25 ng pRL-TK and 100 ng p-(−1.5/+0.1k), followed by incubation with 0.5 μM Dox for 24 hours.

To explore the effect of p53 on the activity of the lnc-Ip53 promoter or the SV40 promoter containing the wild-type or mutant p53RE, cells were co-transfected with 25 ng pRL-TK, 50 ng *firefly* luciferase reporter plasmid [p-(−1.5/+0.1k), p-Δp53RE, pGL3-promoter, p-p53RE-wt or p-p53RE-mut] and 150 ng pCDH-CMV-MCS-EF1-copGFP, pCDH-p53wt or pCDH-p53mut for 48 hours.

To examine the activity of p53 signaling, cells were transfected with 10 nM silnc-Ip53 or NC duplex for 24 hours, followed by co-transfection of 25 ng pRL-TK and 25 ng *firefly* luciferase reporter plasmids containing wild-type (PG13-Luc; Addgene Plasmid #16442, Cambridge, MA, USA) or mutant (MG15-Luc; Addgene Plasmid #16443) p53 binding sites upstream of the *firefly* luciferase gene for another 36 hours.

Electrophoretic mobility shift assay (EMSA) and antibody-supershift assay: Nuclear extracts of SK-HEP-1 cells were prepared as described above. EMSA was performed as previously described[2] using LightShift® Chemiluminescent EMSA kit (Thermo Scientific). Briefly, the biotin-labeled lnc-Ip53 probe, corresponding to the predicted p53RE in the lnc-Ip53 promoter was incubated with nuclear extracts at room temperature (RT) for 30 minutes and subjected to native-polyacrylamide gel electrophoresis (PAGE). For competition assay, 5-fold molar excess of unlabeled p53 consensus binding oligonucleotides were co-incubated with nuclear extracts and labeled probe.

For antibody-supershift assay, nuclear extracts were pre-incubated with 1 μg of anti-p53 antibody (sc-126; Santa Cruz Biotechnology) or isotype-matched IgG (negative control; A7016; Beyotime) at RT for 20 minutes, then added to the binding reaction solution that contained biotin-labeled probe and incubated at RT for another 20 minutes. The sequences of

probes/oligos are listed in Table S2, Supporting Information.

Cell cycle analysis: Cells were treated with a detergent-containing hypotonic solution [Krishan′s reagent: 0.05 mg mL^{-1} propidium iodide (PI), 0.1% sodium citrate, 10 mM NaCl, 0.02 mg mL^{-1} RNase A (2158; TaKaRa), 0.3% NP-40], then analyzed by fluorescence-activated cell sorting (FACS; Gallios, Beckman Coulter, Miami, FL, USA).

For loss-of-function studies, cells were transfected with the indicated siRNAs for 39 hours, then treated with 0.1 μM Dox for 9 hours before FACS analysis. For gain-of-function studies, cells stably expressing lnc-Ip53 and their controls were incubated with 0.1 μM Dox for 29 hours (SK-HEP-1) or 26 hours (U2OS), then resuspended in Krishan′s reagent and incubated at 4 °C overnight before FACS analysis.

Apoptosis analysis: Apoptosis was evaluated by 4′-6′-diamidino-2-phenylindole (DAPI; Sigma-Aldrich) staining for morphology examination, annexin V staining for FACS analysis and immonublotting for cleaved caspase-3. For morphological examination, cells were stained with DAPI and those with condensed or fragmented nuclei were considered as apoptotic cells. At least 500 cells were counted for each sample. Annexin V/PI staining assay was performed using AnnexinV-FITC/PI apoptosis detection kit (Bimake), followed by FACS analysis. Caspase-3 was detected by immunoblotting using antibody against caspase-3 (9662S; CST) for cleaved caspase-3 (17/19 kDa) and pro-caspase-3 (35 kDa).

In loss-of-function studies for spontaneous apoptosis, cells were transfected with the indicated siRNAs for 72 hours before AnnexinV/PI staining as well as DAPI staining and for 68 hours before immunoblotting. In loss-of-function studies for Dox-induced apoptosis, cells were transfected with the indicated siRNAs for 44 hours, then incubated with 1 μM Dox for 28 hours (DAPI) or 24 hours (immunoblotting). For gain-of-function studies, cells stably expressing lnc-Ip53 or their controls were incubated with 1 μM Dox for 44 hours (DAPI) or 42 hours (immunoblotting).

Cell counting assay: Cell counting assay was used to evaluate cell growth. For loss-of-function studies, cells were transfected with the indicated siRNAs for 24 hours, then reseeded (0.5×10^4 for SK-HEP-1, 1×10^4 for U2OS) in a 24-well plate and cultured for 6 days before cell counting. For gain-of-function studies, cells (0.5×10^4 for SK-HEP-1, 1×10^4 for U2OS) stably expressing lnc-Ip53 or their controls were reseeded in a 24-well plate, then cultured for 6 days before cell counting by Countstar (ALIT Life Sciences, Shanghai, China).

Colony formation assay: For loss-of-function studies, cells were transfected with the indicated siRNAs for 24 hours, then reseeded (500 for SK-HEP-1, 1000 for U2OS) in a 6-well plate and maintained in complete medium for 10 days (SK-HEP-1) or 14 days (U2OS). For gain-of-function studies, cells (150 for SK-HEP-1, 500 for U2OS) stably expressing lnc-Ip53 or their controls were reseeded in a 6-well plate and maintained in complete medium for 10 days (SK-HEP-1) or 14 days (U2OS). Colonies were fixed in methanol for 15 minutes, stained with a 0.1% crystal violet solution in 20% methanol for 20 minutes, washed three times with PBS and then counted.

References

[1] W. W. Du, W. Yang, E. Liu, Z. Yang, P. Dhaliwal, B. B. Yang, *Nucleic Acids Res.* **2016**, *44*, 2846.

[2] N. Zhao, S. Li, R. Wang, M. Xiao, Y. Meng, C. Zeng, J. H. Fang, J. Yang, S. M. Zhuang, *Biochim. Biophys. Acta, Gene Regul. Mech.* **2016**, *1859*, 933.

Supplementary figures and legends

Figure S1

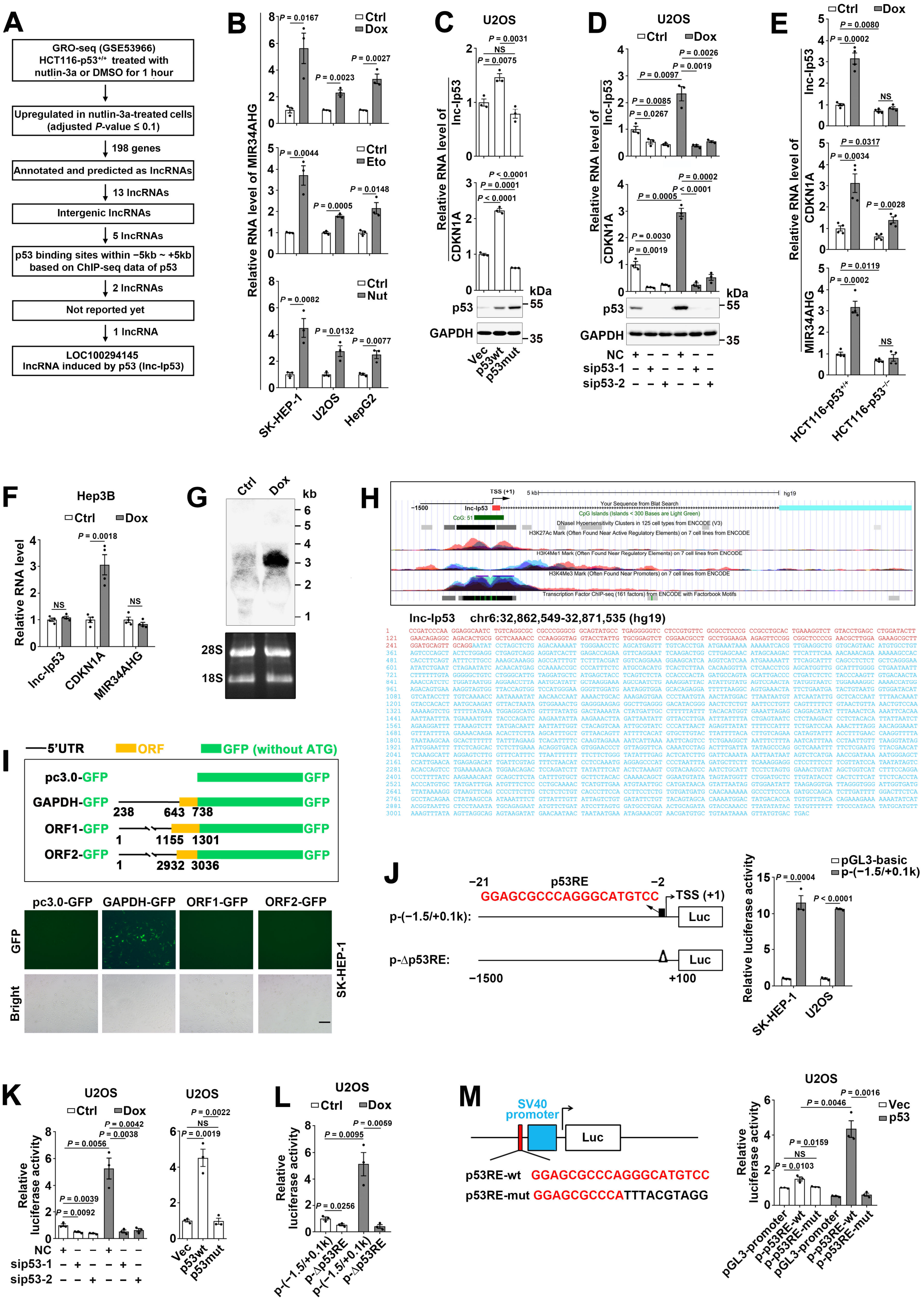

Figure S1. Lnc-Ip53 is a transcriptional target of p53. A) Workflow of the screening procedure to identify p53-regulated lncRNAs. GRO-seq, Global Run-On sequencing. B) Various p53 activators induced MIR34AHG expression. Cells were treated for 12 hours with 0.5 μM Dox, 50 μM etoposide (Eto), 10 μM nutlin-3a (Nut) or vehicle control (Ctrl: PBS as control for Dox, DMSO as control for Eto and Nut). C) Overexpression of wild-type but not mutant p53 increased the lnc-Ip53 level. D) Silencing p53 (sip53) decreased lnc-Ip53 level. E,F) Dox induced lnc-Ip53 expression in p53 wild-type cells but not in p53-null cells. HCT116-p53$^{+/+}$, HCT116-p53$^{-/-}$ (E) and Hep3B (F) cells were treated with PBS (vehicle control, Ctrl), 1.5 μM (HCT116) or 0.5 μM (Hep3B) Dox for 12 hours. G) Detection of cellular lnc-Ip53 by northern blotting. The 28S and 18S rRNAs were used as loading controls. H) The genomic characteristics of potential lnc-Ip53 promoter (*upper*) and the full-length lnc-Ip53 transcript (*lower*). The 5′-end and 3′-end of lnc-Ip53 transcript were identified by 5′RACE and 3′RACE, respectively. The lnc-Ip53 transcript consists of two exons (the first exon in red font and the second in blue font), based on the UCSC Genome Browser. Transcription start site (TSS) is designated as +1 (chr6:32,862,549). I) The lnc-Ip53 transcript had no protein-coding capacity. GAPDH-GFP was used as a positive control and GFP without translation start codon (pc3.0-GFP) as a negative control. Scale bar, 100 μm. J) The −1.5 ~ +0.1k region of lnc-Ip53 showed promoter activity. Schematic diagram (*left*) for *firefly* luciferase reporter plasmids containing −1.5 ~ +0.1k region of lnc-Ip53 is shown. p53RE in the lnc-Ip53 promoter is depicted as closed rectangle and deletion of p53RE is depicted as open triangle. K) sip53 reduced the activity of the lnc-Ip53 promoter (*left*), whereas overexpressing wild-type but not mutant p53 increased its activity (*right*). L) Deletion of p53RE reduced the activity of the lnc-Ip53 promoter. M) p53 enhanced the activity of pGL3-promoter reporter containing wild-type but not mutant p53RE sequence. Schematic diagram (*left*) for pGL3-promoter-based *firefly* luciferase reporter plasmids containing wild-type or mutant p53RE is shown. + or −, cells with (+) or without (−)

the indicated treatment. Data are shown as mean ± SEM of three independent experiments; *P* values were determined by unpaired Student′s *t*-test; NS, not significant.

Figure S2

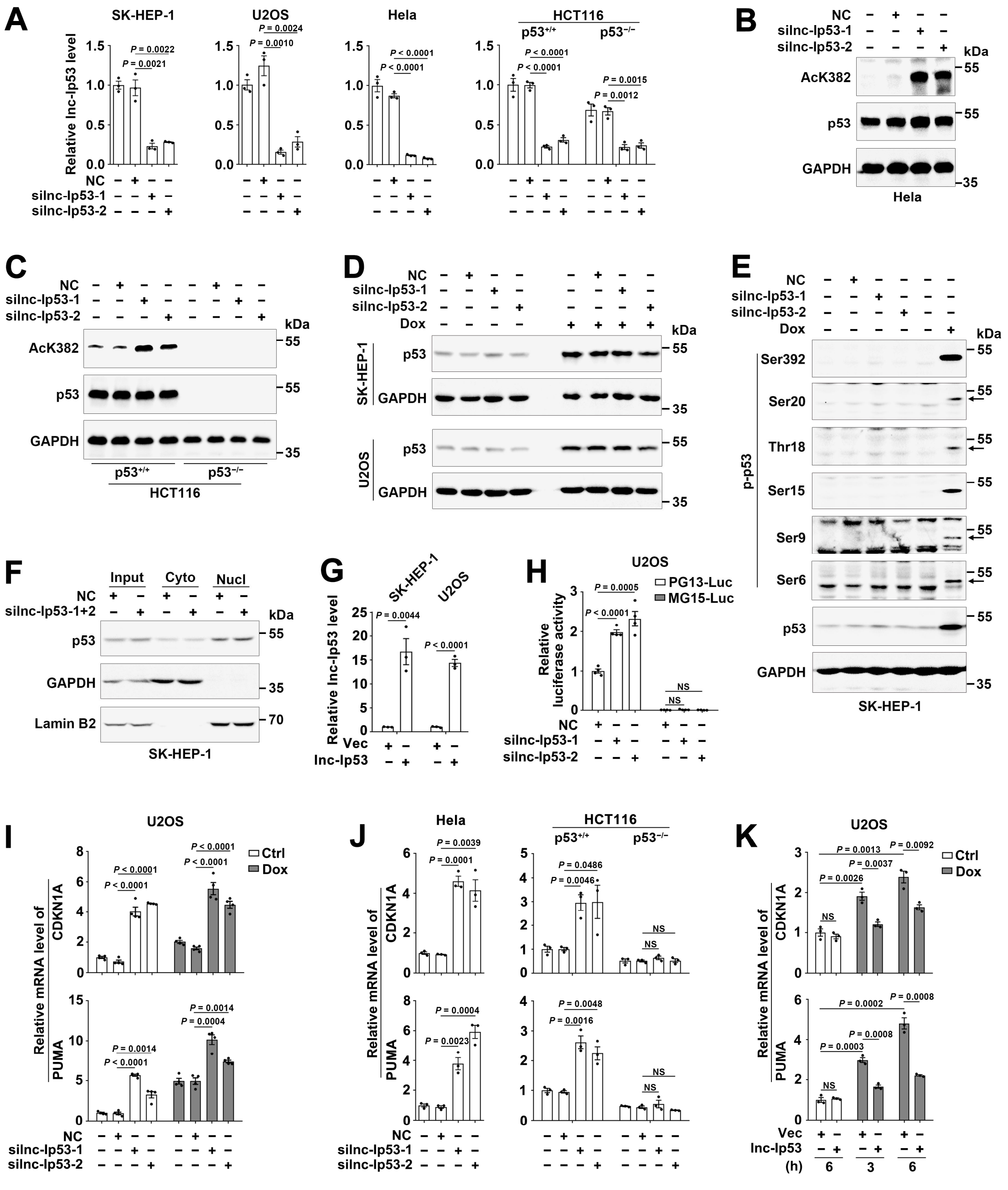

Figure S2. Lnc-Ip53 diminishes the transcriptional activity of p53 by inhibiting its acetylation. A) The RNA level of cellular lnc-Ip53 was reduced by silnc-Ip53. Cells were transfected with the indicated RNA duplexes for 48 hours. NC, negative control. silnc-Ip53-1 and silnc-Ip53-2, siRNA targeting different regions of lnc-Ip53. B,C) Silencing lnc-Ip53 (silnc-Ip53) increased the level of K382-acetylated p53 (AcK-382) in the cells with wild-type p53. Cells were transfected with the indicated RNA duplexes for 42 hours, then incubated with 5 μM SAHA for

6 hours before immunoblotting. D-F) silnc-Ip53 did not affect the protein levels of p53 (D) and phosphorylated p53 (E) and the subcellular localization of p53 (F). For (D,E), cells were transfected with the indicated RNA duplexes for 42 hours, then incubated with PBS or 0.25 μM Dox for 6 hours. The band for the target protein is indicated by arrow. For (F), cells were transfected with the indicated RNA duplexes for 48 hours, followed by subcellular fraction. GAPDH and lamin B2 were used as markers for cytoplasmic (Cyto) and nuclear (Nucl) proteins, respectively. G) Establishment of cell lines stably expressing lnc-Ip53. H) silnc-Ip53 enhanced the luciferase activity of p53-reponsive reporter. I,J) silnc-Ip53 increased the mRNA levels of CDKN1A and PUMA in p53 wild-type cells (U2OS, Hela, HCT116-p53^{+}/$^{+}$) but not in p53-null (HCT116-p53$^{-/-}$) cells. Cells were transfected with the indicated RNA duplexes for 46 hours, then incubated with PBS (Ctrl) or 0.25 μM Dox for 6 hours (I) or transfected with the indicated RNA duplexes for 52 hours (J), followed by qPCR. K) Lnc-Ip53 attenuated the Dox-triggered increase in CDKN1A and PUMA levels. Cells were incubated with PBS for 6 hours or 0.25 μM Dox for 3 or 6 hours. For (G,K), cells stably expressing lnc-Ip53 or control vector (Vec) were used. + or −, cells with (+) or without (−) the indicated treatment. Data are shown as mean ± SEM of three independent experiments; *P* values were determined by unpaired Student′s *t*-test; NS, not significant.

Figure S3

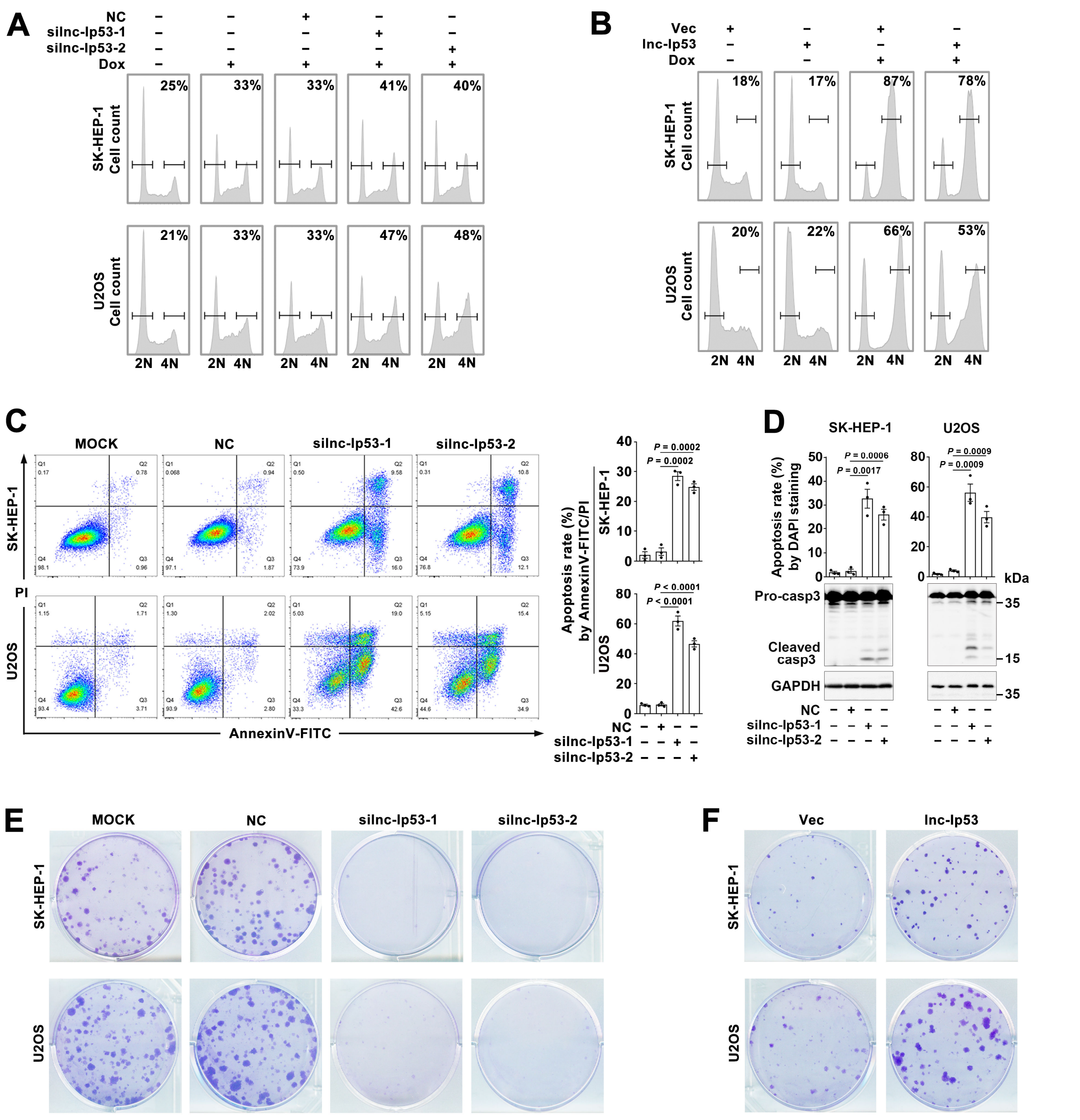

Figure S3. Lnc-Ip53 impairs the p53-mediated cell cycle arrest and apoptosis. A,B) Dox-induced G2/M arrest was promoted by silnc-Ip53 (A) but attenuated by lnc-Ip53 overexpression (B). Representative images for Figure 3A (A) and Figure 3B (B). C,D) silnc-Ip53 promoted spontaneous apoptosis. Cells were transfected with the indicated RNA duplexes for 72 hours before AnnexinV/PI staining (C) and DAPI staining (D) or for 68 hours before immunoblotting (D). E,F) Colony formation was inhibited by silnc-Ip53 (E) but enhanced by

lnc-Ip53 overexpression (F). Representative images of Figure 3E (E) and 3F (F) are shown. + or −, cells with (+) or without (−) the indicated treatment. Data are shown as mean ± SEM of three independent experiments; *P* values were determined by unpaired Student′s *t*-test; NS, not significant.

Figure S4

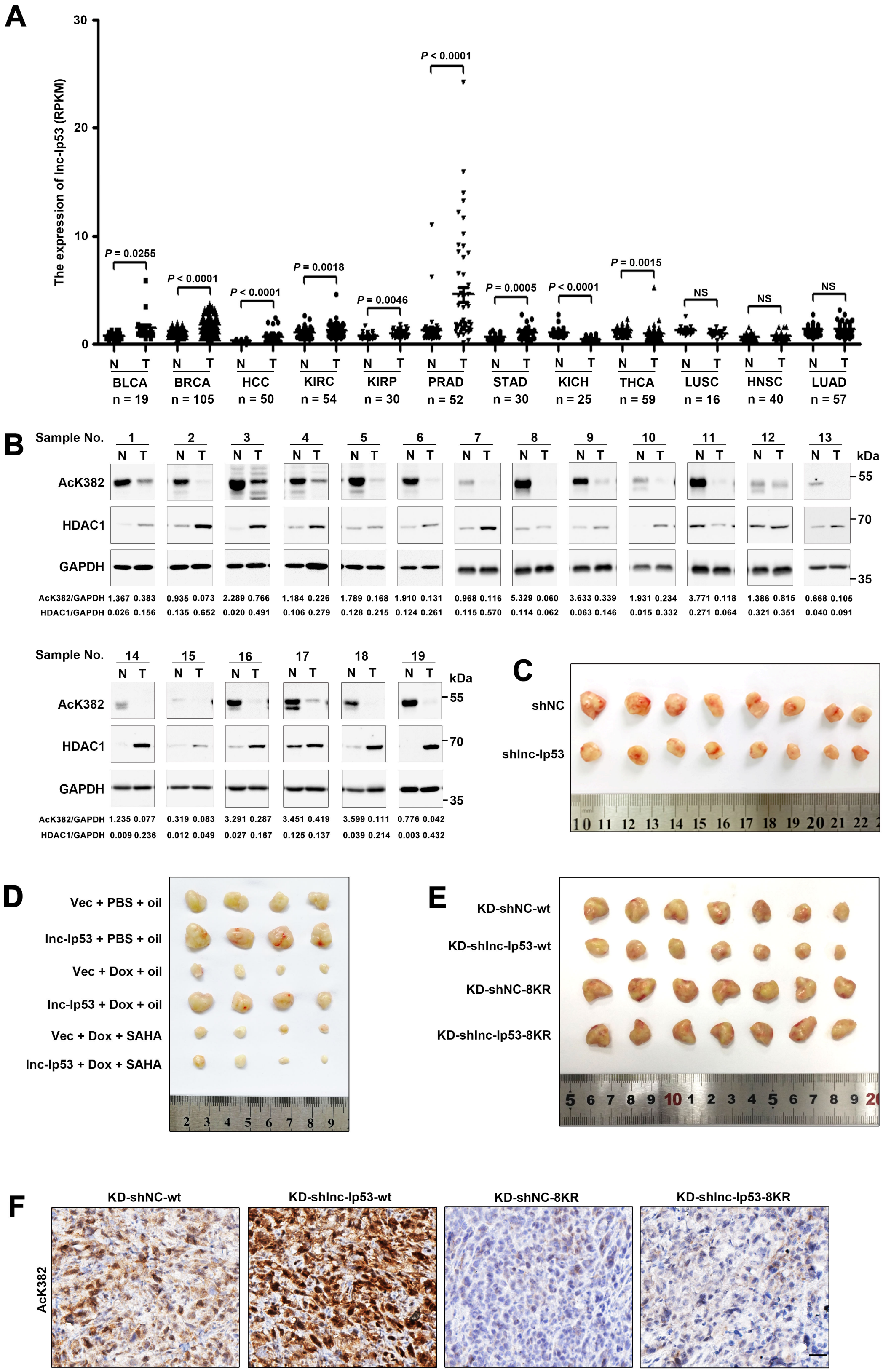

Figure S4. Lnc-Ip53 promotes tumor growth and chemoresistance by impeding p53 acetylation

in vivo. A) Pan-cancer analysis of lnc-Ip53 expression based on TCGA data. The levels of lnc-Ip53 in paired tumor (T) and non-tumor (N) tissues from 12 different cancer types are presented. RPKM, reads per kilobase per million mapped reads. n, the number of examined cases. BLCA, bladder urothelial carcinoma; BRCA, breast invasive carcinoma; HCC, hepatocellular carcinoma; KIRC, kidney renal clear cell carcinoma; KIRP, kidney renal papillary cell carcinoma; PRAD, prostate adenocarcinoma; STAD, stomach adenocarcinoma; KICH, kidney chromophobe; THCA, thyroid carcinoma; LUSC, lung squamous cell carcinoma; HNSC, head and neck squamous cell carcinoma; LUAD, lung adenocarcinoma. Data are expressed as the mean ± SEM; *P* values were determined by paired student's *t* test; NS, not significant. B) The levels of K382-acetylated p53 (AcK382) and HDAC1 were assessed by immunoblotting in 19 paired HCC (T) and adjacent non-tumor liver (N) tissues. C) The xenografts with lnc-Ip53 silencing displayed reduced tumor growth. The xenografts of SK-shNC and SK-shlnc-Ip53 cells were dissected at 27 days after implantation. D) The xenografts with lnc-Ip53 overexpression showed enhanced tumor growth and chemoresistance. The xenografts of SK-Vec and SK-lnc-Ip53 were injected with vehicle (PBS and corn oil), Dox, or combined Dox and SAHA, and the xenografts were dissected 30 days after implantation. E,F) Silencing lnc-Ip53 repressed tumor growth (E) and decreased the levels of acetylated-p53 (F) in xenografts with wild-type p53, but had no effect on those with acetylation-resistant p53 (8KR). The xenografts of SK-KD-shNC-wt, SK-KD-shlnc-Ip53-wt, SK-KD-shNC-8KR or SK-KD-shlnc-Ip53-8KR cells were dissected 30 days after implantation. For (C-E), photographs of dissected tumors are shown. For (F), the levels of K382-acetylated p53 (AcK-382) in xenografts were detected by IHC and representative images for Figure 4L are shown. Scale bar: 25 μm.

Figure S5

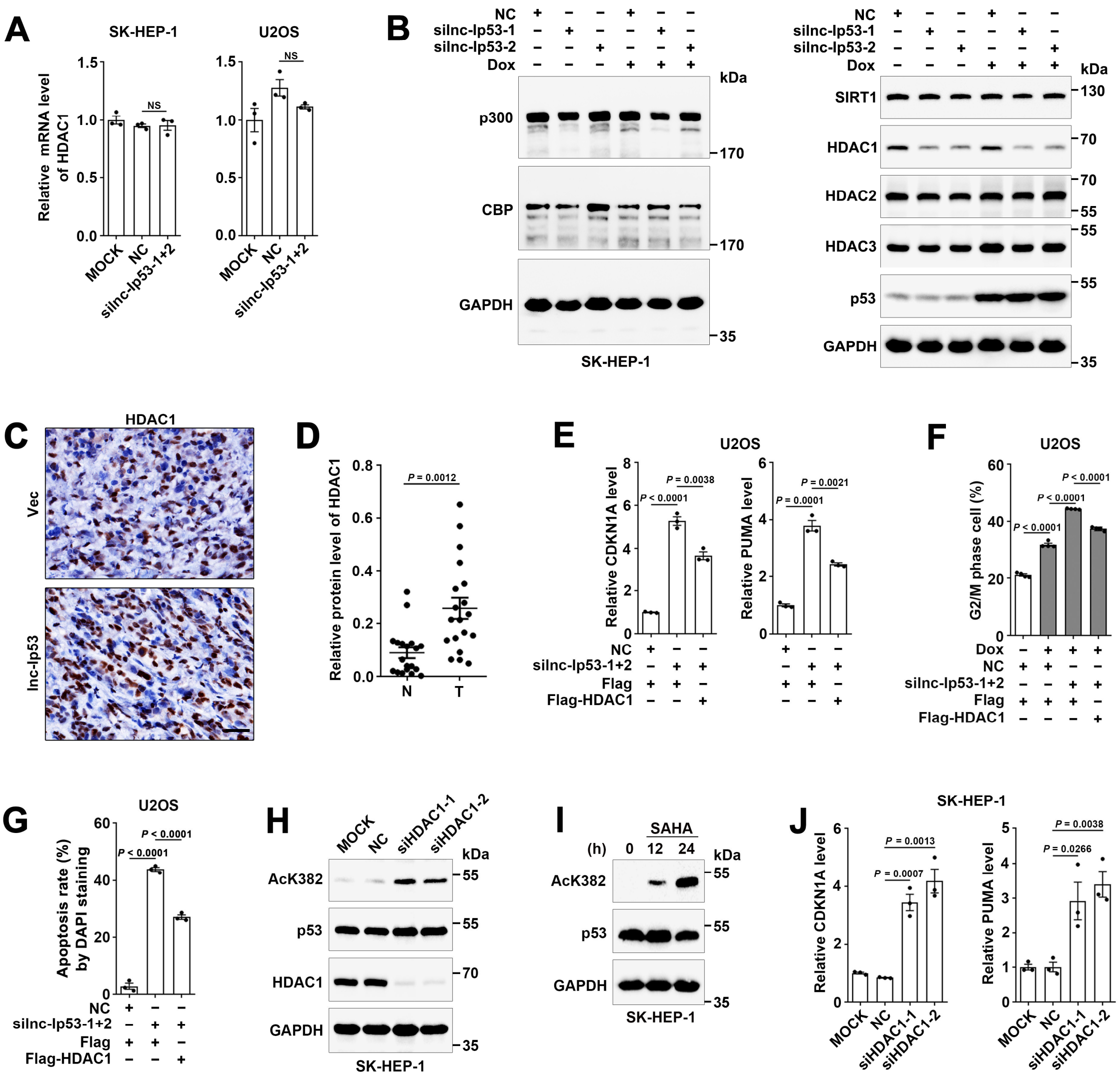

Figure S5. Lnc-Ip53 inhibits p53 acetylation/activity by increasing HDAC1 level. A) silnc-Ip53 didn't reduce the mRNA level of HDAC1. Cells were transfected with the indicated RNA duplexes for 48 hours before qPCR. MOCK, cells exposed to Lipofectamine RNAiMAX but not RNA duplexes. B) Effects of lnc-Ip53 silencing on the levels of key regulators of p53 acetylation. Cells were transfected with the indicated RNA duplexes for 36 hours, then incubated with PBS or 0.5 μM Dox for 12 hours before immunoblotting. C) The xenografts with lnc-Ip53 overexpression showed increased HDAC1 level. The xenografts from Figure S4D were analyzed by IHC for HDAC1. Representative images for Figure 5C are shown. Scale

bar: 25 μm. D) The HDAC1 level increased in human HCC. The levels of HDAC1 in HCC (T) and adjacent non-tumor liver (N) tissues were derived from Figure S4B. E) HDAC1 overexpression abrogated silnc-Ip53-stimulated CDKN1A/PUMA expression. Cells stably expressing Flag or Flag-HDAC1 were transfected with the indicated RNA duplexes for 52 hours before qPCR. F,G) HDAC1 overexpression abrogated silnc-Ip53-induced G2/M arrest (F) and apoptosis (G). Cells stably expressing Flag or Flag-HDAC1 were transfected with the indicated RNA duplexes for 39 hours and then incubated with 0.1 μM Dox for 9 hours before cell cycle analysis (F), or transfected with the indicated RNA duplexes for 72 hours before DAPI staining (G). H) Silencing HDAC1 (siHDAC1) by RNA interference promoted p53 acetylation. Cells were transfected with the indicated RNA duplexes for 48 hours before immunoblotting. I) Repression of HDAC1 activity by HDAC inhibitor SAHA promoted p53 acetylation. Cells were incubated with 5 μM SAHA for the indicated time before immunoblotting. J) siHDAC1 increased the levels of CDKN1A/PUMA. Cells were transfected with the indicated RNA duplexes for 52 hours before qPCR. + or −, cells with (+) or without (−) the indicated treatment. Data are shown as mean ± SEM of three independent experiments; *P* values were determined by unpaired (A,E-G,J) or paired (D) Student's *t*-test; NS, not significant.

Figure S6

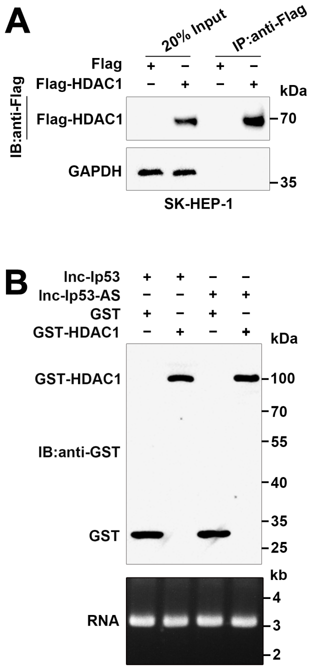

Figure S6. The immunoprecipitation (IP) efficiency of anti-Flag affinity in RIP assay for Figure 6E (A) and the input of protein and RNA in GST pulldown assay for Figure 6F (B). For (A), cells stably expressing Flag or Flag-HDAC1 were subjected to RIP assay using anti-Flag affinity gel, followed by immunoblotting (IB) using anti-Flag antibody. GAPDH was used as a negative control. For (B), GST or GST-HDAC1 proteins were incubated with lnc-Ip53 or lnc-Ip53-AS, followed by immunoblotting using anti-GST antibody or detection of RNA. + or −, cells with (+) or without (−) the indicated treatment.

Figure S7

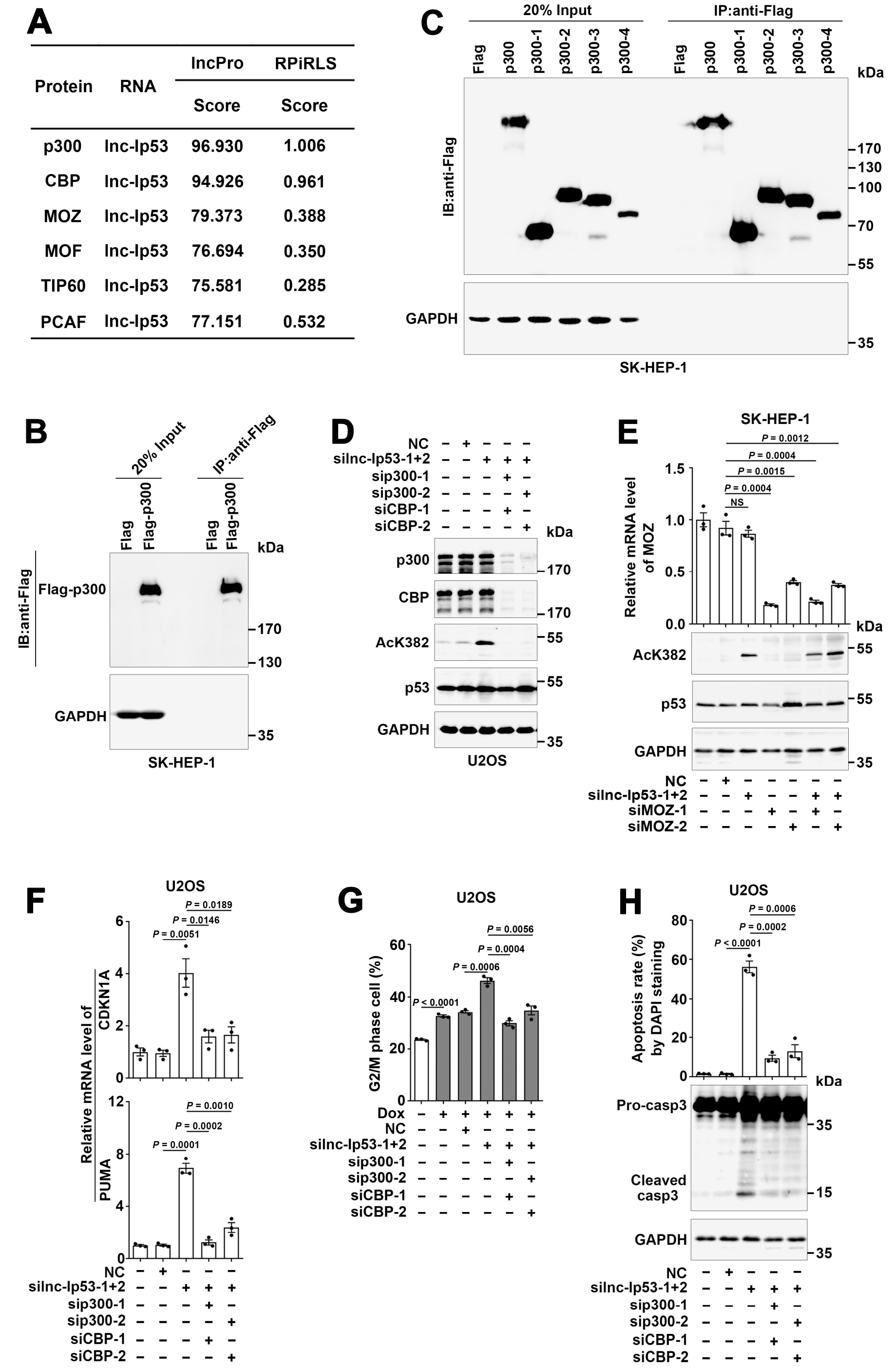

Figure S7. Lnc-Ip53 associates with p300 and attenuates its activity to acetylate p53. A) p300/CBP were predicted to possess the highest interaction strength with lnc-Ip53. The potential interaction between lnc-Ip53 and histone acetyltransferases was predicted using lncPro (http://bioinfo.bjmu.edu.cn/lncpro/) and RPiRLS (http://bmc.med.stu.edu.cn/RPiRLS).

Greater score suggests higher interaction strength. B,C) The immunoprecipitation efficiency of anti-Flag affinity in RIP assays for Figure 7A (B) and 7B (C). Cells stably expressing Flag or Flag-tagged p300 fragments were subjected to RIP assays using anti-Flag affinity gel, followed by immunoblotting (IB) using anti-Flag antibody. GAPDH was used as a negative control. D) Silencing p300/CBP (sip300/CBP) attenuated silnc-Ip53-stimulated p53 acetylation. Cells were transfected with the indicated RNA duplexes for 42 hours, then incubated with 5 μM SAHA for 6 hours before immunoblotting for K382-acetylated p53 (AcK382). E) Silencing MOZ (siMOZ) didn′t affect silnc-Ip53-stimulated p53 acetylation. Cells were transfected with the indicated RNA duplexes for 48 hours before qPCR analysis for MOZ (*upper*), or for 42 hours, followed by incubation with 5 μM SAHA for 6 hours before immunoblotting (*lower*). F-H) Silencing p300/CBP abrogated the stimulatory effect of silnc-Ip53 on CDKN1A/PUMA expression (F), G2/M arrest (G) and apoptosis (H). Cells were transfected with the indicated RNA duplexes for 52 hours before qPCR (F), or for 39 h and then incubated with 0.1 μM Dox for 9 hours before cell cycle analysis (G), or transfected with the indicated RNA duplexes for 72 hours before DAPI staining or 68 hours before immunoblotting (H). + or −, cells with (+) or without (−) the indicated treatment. Data are shown as mean ± SEM of three independent experiments; *P* values were determined by unpaired Student′s *t*-test; NS, not significant.

Supplementary tables

Table S1. The list of candidate lncRNAs[a)]

Gene Name	FC[b)]	Intergenic[c)]	p53 binding site[d)]	Reported[e)]
LOC283761	8.04	YES	NO	NO
RNF157-AS1	4.36	NO	NO	NO
KRT18P55	3.88	NO	NO	NO
LOC643401	3.87	YES	YES	YES
LOC100506343	3.81	NO	YES	NO
LINC00158	3.43	YES	NO	NO
LINC00589	2.86	YES	NO	NO
FLJ44511	2.72	NO	YES	NO
LOC284080	2.50	NO	YES	YES
TRAF3IP2-AS1	2.48	NO	YES	NO
SMAD5-AS1	2.44	NO	YES	NO
**LOC100294145 （lnc-Ip53）**	**2.14**	**YES**	**YES**	**NO**
LOC284385	2.10	NO	YES	NO

[a)]GRO-seq data was obtained from GEO database (accession no. GSE53966). Only non-coding genes predicted by CPAT are included.

[b)]FC, fold change. The level of lncRNA in nutlin-3a-treated HCT116-p53^{+}/$^{+}$ cells relative to that in DMSO-treated HCT116-p53^{+}/$^{+}$ cells.

[c)]LncRNA is (YES) or is not (NO) located in intergenic region.

[d)]There is (YES) or there is no (NO) p53 binding sites within the −5kb ~ +5kb region (transcription start site denoted as +1) of lncRNA based on ChIP-seq data of p53 from GEO database (accession no. GSM1133486).

[e)]LncRNA has (YES) or has not (NO) been reported.

Table S2. Sequences of RNA and DNA oligonucleotides

Name	Sense Strand/Sense Primer (5'-3')	Antisense Strand/Antisense Primer (5'-3')
**siRNA duplexes**		
silnc-Ip53-1	GUUGAAGGCUGGUGCAGUAdTdT	UACUGCACCAGCCUUCAACdGdT
silnc-Ip53-2	CCAUUGAACAUGAGAGACAdTdT	UGUCUCUCAUGUUCAAUGGdAdG
siCBP-1	CCAAUGUGUUAGAAGAGAGCAUUAAdTdT	UUAAUGCUCUCUUCUAACACAUUGGdTdT
siCBP-2	GACAUCCCGAGUCUAUAAGdTdT	CUUAUAGACUCGGGAUGUCdTdT
sip300-1	GCUUAGAUGAUGAGAGCAA dTdT	UUGCUCUCAUCAUCUAAGC dTdT
sip300-2	CGGAAAACAUCACGGGUAUdTdT	AUACCCGUGAUGUUUUCCGdGdT
sip53-1	UCCAGAUGAAGCUCCCAGAdTdT	UCUGGGAGCUUCAUCUGGAdCdC
sip53-2	CCACCAUCCACUACAACUACAUGdTdT	CAUGUAGUUGUAGUGGAUGGUGGdTdA
siMOZ-1	UUAAUCUGCACUUCAGAGCCUCAGGdTdT	CCUGAGGCUCUGAAGUGCAGAUUAAdTdT
siMOZ-2	GGUCACUCAGCAGAUGGCCAGCAUGdTdT	CAUGCUGGCCAUCUGCUGAGUGACCdTdT
siHDAC1-1	GCGACUGUUUGAGAACCUUdTdT	AAGGUUCUCAAACAGUCGCdTdT
siHDAC1-2	GGGAUCGGUUAGGUUGCUUdTdT	AAGCAACCUA ACCGAUCCCdTdT
NC	UGAAUUAGAUGGCGAUGUUdTdT	AACAUCGCCAUCUAAUUCAdTdT
**Primers for qPCR**		
lnc-Ip53	ACAAGCCAATCTGCATTTCC	TTTGCAGGGGAGAAACATTC
CDKN1A	GACCATGTGGACCTGTCACT	GATTAGGGCTTCCTCTTGGA
MIR34AHG	GGAGATTTTGCGGGAGTCCT	AGTTCCCGCCTCCTTCTTTG
PUMA	AGAGCAGGGCAGGAAGTAAC	GGCTGAGGACCACAAATCTG
HDAC1	TAAATTCTTGCGCTCCATCC	AACAGGCCATCGAATACTGG
MOZ	CGAGAAAAGAAGCCAGAGGA	GATCTCGACAGGAGCTGCAT
18S rRNA	GGTGGATCACTCGGCTCGT	GCAAGTGCGTTCGAAGTGTC
U6	CTCGCTTCGGCAGCACA	AACGCTTCACGAATTTGCGT
GAPDH	GAGTCAACGGATTTGGTCGT	GACAAGCTTCCCGTTCTCAG
**Probes for EMSA**		
lnc-Ip53 (p53RE)	GAGCTGGAGCGCCCAGGGCATGTCCGCCGA	TCGGCGGACATGCCCTGGGCGCTCCAGCTC
p53 consensus	TACAGAACATGTCTAAGCATGCTGGGGACT	AGTCCCCAGCATGCTTAGACATGTTCTGTA
non-specific oligo	TACACCTTCGCAGATCTAGCTGAGGGGACT	AGTCCCCTCAGCTAGATCTGCGAAGGTGTA
**Primers for ChIP**		
lnc-Ip53	CTCTGCCTACAGCGTTCCTC	GGCGGGAGGC GCGAACAC
CDKN1A	CCCACAGCAGAGGAGAAAGAA	CTGGAAATCTCTGCCCAGACA
PUMA	GCGAGACTGTGGCCTTGTGT	CGTTCCAGGGTCCACAAAGT
Negative (GAPDH)	TACTAGCGGTTTTACGGGCGCACGT	TCGAACAGGAGGAGCAGAGAGCGAA
**Probes for northern blotting**		
lnc-Ip53	AGAGAGTTCCCGTATGTCCCCTCAAGCCCTCTTCCCTCCAGTTTCCACTAT	

Table S2. Sequences of RNA and DNA oligonucleotides (Continued)

Name	Sense Strand/Sense Primer (5'-3')	Antisense Strand/Antisense Primer (5'-3')
**Primers for cloning (Restriction enzyme sites were underlined; homologous arm was indicated by lowercase letters; initiation codon and stop codon were indicated by bold letters.)**		
**Cloning into pCDH**		
lnc-Ip53	atagaagattctagagctagcCCGATCCCAAGGAGGCAATC	atccgatttaaattcgaattcGTCAGTCACATAACTTTTATTACA
Ip53-core	atagaagattctagagctagcAACAGGTGACAGTGGAACCC	atccgatttaaattcgaattcGGAGAAACATTCAAATCATAGCAC
△Ip53-core	CTCTCTTGAACCTGCAAAACTCATGTTGAA	GTTTTGCAGGTTCAAGAGAGTGCTGAGAAA
p53wt	CCGGAATTCGCCACC**ATG**GAGGAGCCGCAGTCAGA	CGCGGATCC**TCA**GTCTGAGTCAGGCCCTT
p53mut	AGGTTGTGAGGCACTGCC	GGCAGTGCCTCACAACCT
**Cloning into pCDH-Flag(N)**		
HDAC1	ggatgacgatgacaagctcgagGCGCAGACGCAGGGCACC	gcggatccgatttaaattcgaattc**TCA**GGCCAACTTGACCTCCT
p300	ggatgacgatgacaagctcgagGCCGAGAATGTGGTGGAAC	gcggatccgatttaaattcgaattc**CTA**GTGTATGTCTAGTGTAC
p300-1	ggatgacgatgacaagctcgagGCCGAGAATGTGGTGGAAC	gcggatccgatttaaattcgaattc**CTA**TCCAGTAGTGGATGGTTG
p300-2	GgatgacgatgacaagctcgagACTACTGGAATTCGGAAAC	gcggatccgatttaaattcgaattc**CTA**GTAACTGTAATAAGTGGCATC
p300-3	ggatgacgatgacaagctcgagCAGAACAGGTATCATTTCTG	gcggatccgatttaaattcgaattc**CTA**CTGCCGGAGCTTCTGCTT
p300-4	ggatgacgatgacaagctcgagCAACAGCTGCAGCACCGA	gcggatccgatttaaattcgaattc**CTA**GTGTATGTCTAGTGTAC
**Cloning into pGEX-6p-1**		
HDAC1	ttccaggggcccctgggatccGCGCAGACGCAGGGCACC	gtcacgatgcggccgctcgag**TCA**GGCCAACTTGACCTCCT
HDAC1/2-150aa	ttccaggggcccctgggatccGCGCAGACGCAGGGCAC	gtcacgatgcggccgctcgag**TCA**GAAGCCAGATGCCTCGGA
HDAC1/151-250aa	ttccaggggcccctgggatccTGTTACGTCAATGATATCGTC	gtcacgatgcggccgctcgag**TCA**CTCCATTACTTTGGACATGAC
HDAC1/251-386aa	ttccaggggcccctgggatccATGTTCCAGCCTAGTGCG	gtcacgatgcggccgctcgag**TCA**CTCAGGAATCGCCTGCAT
HDAC1/387-482aa	ttccaggggcccctgggatccGACGCCATCCCTGAGGAG	gtcacgatgcggccgctcgag**TCA**GGCCAACTTGACCTCCTC
p53	ttccaggggcccctgggatccGAGGAGCCGCAGTCAGA	gtcacgatgcggccgctcgag**TCA**GTCTGAGTCAGGCCCTT
**Cloning into pGL3-basic**		
p-(−1.5/+0.1k)	CTAGCTAGCGACCTGCAGTTTCAAGCCAA	CCCAAGCTTAGACCTTTCAGTGCAGGC
p-△p53RE	CTGAGGCGCGCGGAGCTGCCGATCCCAAGGAGGC	GCCTCCTTGGGATCGGCAGCTCCGCGCGCCTCAG
**Cloning into pGL3-promoter**		
p-p53RE-wt	CTAGCGGAGCGCCCAGGGCATGTCCA	GATCTGGACATGCCCTGGGCGCTCCG
p-p53RE-mut	CTAGCGGAGCGCCCATTTACGTAGGA	GATCTCCTACGTAAATGGGCGCTCCG

Table S2. Sequences of RNA and DNA oligonucleotides (Continued)

Name	Sense Strand/Sense Primer (5'-3')	Antisense Strand/Antisense Primer (5'-3')
**Cloning into pcDNA3.0**		
GFP	ccgccagtgtgctggaattcGTGAGCAAGGGCGAGGA	atgcatgctcgagcggccgc**TCA**CTTGTACAGCTCGTCC
GAPDH-GFP	ccgccagtgtgctggaattcCTGTTTCATCCAAGCGTGTAA	AGCTCCTCGCCCTTGCTCACGTTAAAAGCAGCCCTGGTGA
ORF1-GFP	ccgccagtgtgctggaattcCCGATCCCAAGGAGGCAATC	AGCTCCTCGCCCTTGCTCACCAGAAAAACTGAACAGG
ORF2-GFP	ccgccagtgtgctggaattcCCGATCCCAAGGAGGCAATC	AGCTCCTCGCCCTTGCTCACTTGTTCATATCTTACTC
**Cloning into pCDH**		
pCDH-Flag(N)	TGCTCTAGAGCCACC**ATG**GACTACAAAGACCATGAC	CCGGAATTCGATATC**CTA**CTCGAGCTTGTCATCGTCATCCTTG
**Primers for RACE**		
5’RACE-GSP1	GCACCAGCCTTCAACGTGAT	
5’RACE-GSP2	CCTCTGTTCAAGTATCCAGGCT	
AUAP	GGCCACGCGTCGACTAGTAC	
3’RACE-GSP1	ACAAGCCAATCTGCATTTCC	
3’RACE-GSP2	CCAGATGCCAGCATCTTGATT	
**Primers for *in vitro* transcription (The sequence of T7 promoter was underlined)**		
lnc-Ip53	ATTAATACGACTCACTATAGGGCCGATCCCAAGGAGGCAATC	GTCAGTCACATAACTTTTATT
lnc-Ip53-AS	CCGATCCCAA GGAGGCAATC	ATTAATACGACTCACTATAGGGGTCAGTCACATAACTTTTATT
1-2500nt	ATTAATACGACTCACTATAGGGCCGATCCCAAGGAGGCAATC	AGGTATACAAGAGCATCCAGAA
1-2000nt	ATTAATACGACTCACTATAGGGCCGATCCCAAGGAGGCAATC	TAATCAAAGTCTAGGATTTGTGAAC
1-1500nt	ATTAATACGACTCACTATAGGGCCGATCCCAAGGAGGCAATC	AATATTAATCTAAGTTTCTTTAATATTC
1-1000nt	ATTAATACGACTCACTATAGGGCCGATCCCAAGGAGGCAATC	TTCCCATGGACCACTGGTAA
1-500nt	ATTAATACGACTCACTATAGGGCCGATCCCAAGGAGGCAATC	GGCAAGAAATACTGAAGGTGGT
1950-2550nt	ATTAATACGACTCACTATAGGGAACAGGTGACAGTGGAACCC	GGAGAAACATTCAAATCATAGCAC
1950-2350nt	ATTAATACGACTCACTATAGGGAACAGGTGACAGTGGAACCC	GGCTTGTACGACTTTAGAGTATG
2150-2550nt	ATTAATACGACTCACTATAGGGAAATGGGAGCTCCATTGAACA	GGAGAAACATTCAAATCATAGCAC
